# Carbon Nanoparticle Oxidation by NO_2_ and O_2_: Chemical Kinetics and Reaction Pathways

**DOI:** 10.1002/anie.202413325

**Published:** 2024-11-26

**Authors:** Thomas Berkemeier, Ulrich Pöschl

**Affiliations:** ^1^ Multiphase Chemistry Department Max Planck Institute for Chemistry Hahn-Meitner-Weg 1 55128 Mainz Germany

**Keywords:** chemical kinetics, heterogeneous chemistry, soot, particles, carbon capture

## Abstract

Carbon nanoparticle interactions with gases are central to many environmental and technical processes, but the underlying reaction kinetics and mechanisms are not well understood. Here, we investigate the oxidation and gasification of carbon nanoparticles by NO_2_ and O_2_ under combustion exhaust conditions. We build on a comprehensive experimental data set and use a kinetic multilayer model (KM‐GAP‐CARBON) to trace the uptake and release of gas molecules alongside the temporal evolution of particle size and surface composition. The experimental results are captured by a model mechanism that involves different types of carbon atoms (edge/plane‐like) and the formation of a reactive oxygen intermediate (activated CO complex) as the rate‐limiting step. A transition between distinct chemical regimes driven by NO_2_ at lower temperatures and O_2_ at higher temperatures is reflected by an increase in the observable activation energy from ∼ 60 kJ/mol to ∼ 130 kJ/mol. We derive energy profiles for three alternative reaction pathways that involve uni‐ or bimolecular decomposition of reactive oxygen intermediates.

## Graphical abstract

Using a kinetic multilayer model and comprehensive experimental data, this study investigates the oxidation of carbon nanoparticles by NO_2_ and O_2_ under combustion exhaust conditions, going beyond traditional shrinking core modelling approaches. The study develops a molecular picture of the reaction system, including a reference chemical reaction mechanisms and energy profiles for three alternative reaction pathways.

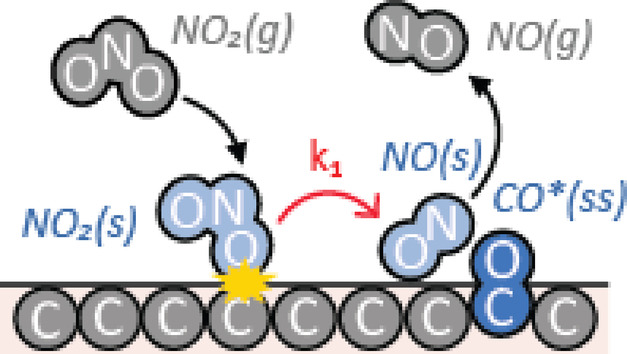

Heterogeneous reactions and multiphase processes between solids, liquids and gases are important in many areas of chemical technology as well as life, health, Earth and climate sciences.[^1^, ^2^, ^3^, ^4^, ^5^] The chemistry of elemental carbon surfaces is of great technological importance. In combustion engineering, the goal is often to minimize the formation of soot particles, e.g., through engine design or development of new fuels and fuel‐blends.^[6]^ The reverse process, the gasification of carbonaceous materials and the consumption of finely dispersed particles, were studied intensively in the development of coal conversion technologies and soot particle filters.[^7^, ^8^] These reactions are traditionally described using shrinking core models,[^9^, ^10^] where structural parameters are incorporated into empirical reaction rate constants, or pore growth models, where structural parameters are incorporated into the reaction scheme.[^11^, ^12^] For a more detailed molecular description of interfacial processes in aerosol kinetics and related gas‐particle‐interactions, a comprehensive framework with universally applicable rate equations[^13^, ^14^] and a variety of multilayer models[^15^, ^16^, ^17^, ^18^, ^19^, ^20^, ^21^, ^22^, ^23^, ^24^, ^25^] have been developed during recent years. These models can be used in combination with global optimization and ensemble techniques to derive kinetic parameters and quantify their uncertainty.[^26^, ^27^]

For the high‐temperature oxidation and gasification of soot particles and related carbonaceous materials by nitrogen oxides and oxygen under conditions relevant for modern diesel engine exhaust and regenerating particle filter systems, Messerer et al.^[28]^ performed comprehensive experimental investigations and analyzed the measurement results with mass‐based pseudo‐first‐order rate coefficients and a traditional shrinking core model. In this study, we take a new approach based on recent developments in multiphase chemical kinetics modelling and re‐analyze the results and data of Messerer et al. with a newly developed kinetic multilayer model (KM‐GAP‐CARBON) to gain insights in the molecular processes and rate‐limiting steps involved in this complex heterogeneous reaction.

Figure 1 schematically depicts the reference chemical mechanism applied in this study (Eqs. R1–R5) to analyze and reproduce the measurement data of high‐temperature oxidation and gasification of carbon nanoparticles by NO_2_ and O_2_ as reported by Messerer et al.^[28]^ This mechanism integrates and builds on related earlier studies investigating the oxidation of carbon nanoparticles, soot, and related carbonaceous materials.[^29^, ^30^, ^31^, ^32^, ^33^, ^34^] It involves Langmuir Hinshelwood‐type heterogeneous reaction kinetics of physisorbed and chemisorbed surface species as established by Ergun^[35]^ and others for the reaction of CO_2_ with activated charcoal and graphites in the Boudouard reaction and applied in many kinetic models of related reaction systems.[^36^, ^37^, ^38^] Specifically, carbon atoms at the particle surface react with physisorbed molecules of NO_2_ (R1) or O_2_ (R2) to form an activated surface complex (CO*) that can be regarded as a chemisorbed oxygen atom, long‐lived reactive oxygen intermediate (ROI),^[39]^ or oxygen‐containing surface functional group (carbonyl).[^31^, ^40^, ^41^] The activated complex (CO*) can undergo a bimolecular second‐order reaction with NO_2_ (R3) and O_2_ (R4) to form CO_2_.[^29^, ^33^, ^38^, ^42^] Alternatively, CO* can undergo a unimolecular, first‐order reaction forming physisorbed CO (R5.
(R1a)

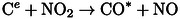



(R1b)

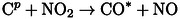



(R2a)

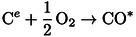



(R2b)

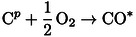



(R3)

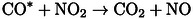



(R4)

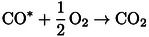

(R5) $${\mathrm{CO}}^{*}\to \mathrm{CO}$$

**Figure 1:**
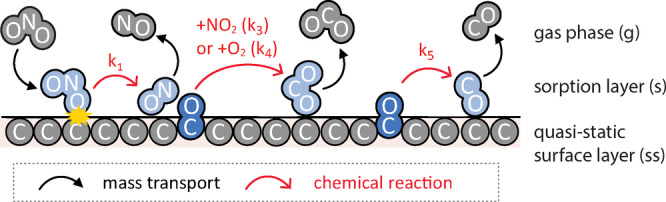
Reference chemical mechanism applied in the kinetic multilayer model of gas‐particle interactions for multiphase chemistry of carbon nanoparticles (KM‐GAP‐CARBON). Carbon atoms at the particle surface react with physisorbed NO_2_ molecules (R1) to form an activated surface complex (CO*, dark blue) that can be further oxidized by NO_2_ or O_2_ to form physisorbed CO_2_ (R3, R4), or undergo a unimolecular transformation into physisorbed CO (R5). Physisorbed species marked in light blue occupy sorption sites on the surface (sorption layer, s), while chemisorbed species marked in dark blue are covalently bound to the underlying particle material (quasi‐static surface layer, ss). For simplicity, the reaction of O_2_ with carbon (R2) is not displayed in this schematic.

Here, the chemical structure and heterogeneity of soot is approximately described by distinguishing two types of carbon atoms that differ in their reactivity with surface‐adsorbed oxidants (NO_2_ and O_2_) and reflect the co‐existence of ordered (graphitic) and disordered (amorphous) structures,[^43^, ^44^, ^45^, ^46^] which are sometimes also characterized and designated as sp^2^ or sp^3^ hybridized carbon atoms, respectively.[^47^, ^48^] In analogy to the nanostructure of graphite, graphenes or PAH, we adopt the terminology of edge‐like carbon atoms (C^*e*^) and basal plane‐like carbon atoms (C^*p*^),[^49^, ^50^, ^51^] whereby the initial oxidation of C^*e*^ atoms or less‐graphitized nanostructures is known to occur faster than that of C^*p*^ atoms or more graphitized nanostructures.[^46^, ^49^, ^52^, ^53^] Details on the applied kinetic multilayer model of gas‐particle interactions in the oxidation of carbon (KM‐GAP‐CARBON) and the determination of rate parameters by global optimization using a Monte Carlo Genetic Algorithm,^[26]^ are provided in the Supporting Information.

Figure 2 shows experimental data and model simulations of the oxidation and gasification of carbon nanoparticles by NO_2_ and O_2_ under reaction conditions that are characteristic for diesel engine exhaust and particle traps (150 ppm NO_2_, 10 % O_2_ and 3 % H_2_O; *T*=548–723 K). The temporal evolution of normalized carbon particle mass (*m*_C_/$m_{C,0}$ , Figure 2a) exhibits a near‐exponential decay that is well described by the model, which captures also the observed temperature dependence of the molar ratio of CO and CO_2_ in the reactor outflow (Figure 2b). The relative increase of CO with increasing temperature can be explained by the decrease of desorption lifetimes and steady‐state surface concentrations of oxidants, which in turn leads to less bimolecular oxidation of CO* to CO_2_ and more first‐order decomposition forming CO. These results are consistent with earlier experimental investigations and mechanistic analyses.^[54]^

**Figure 2:**
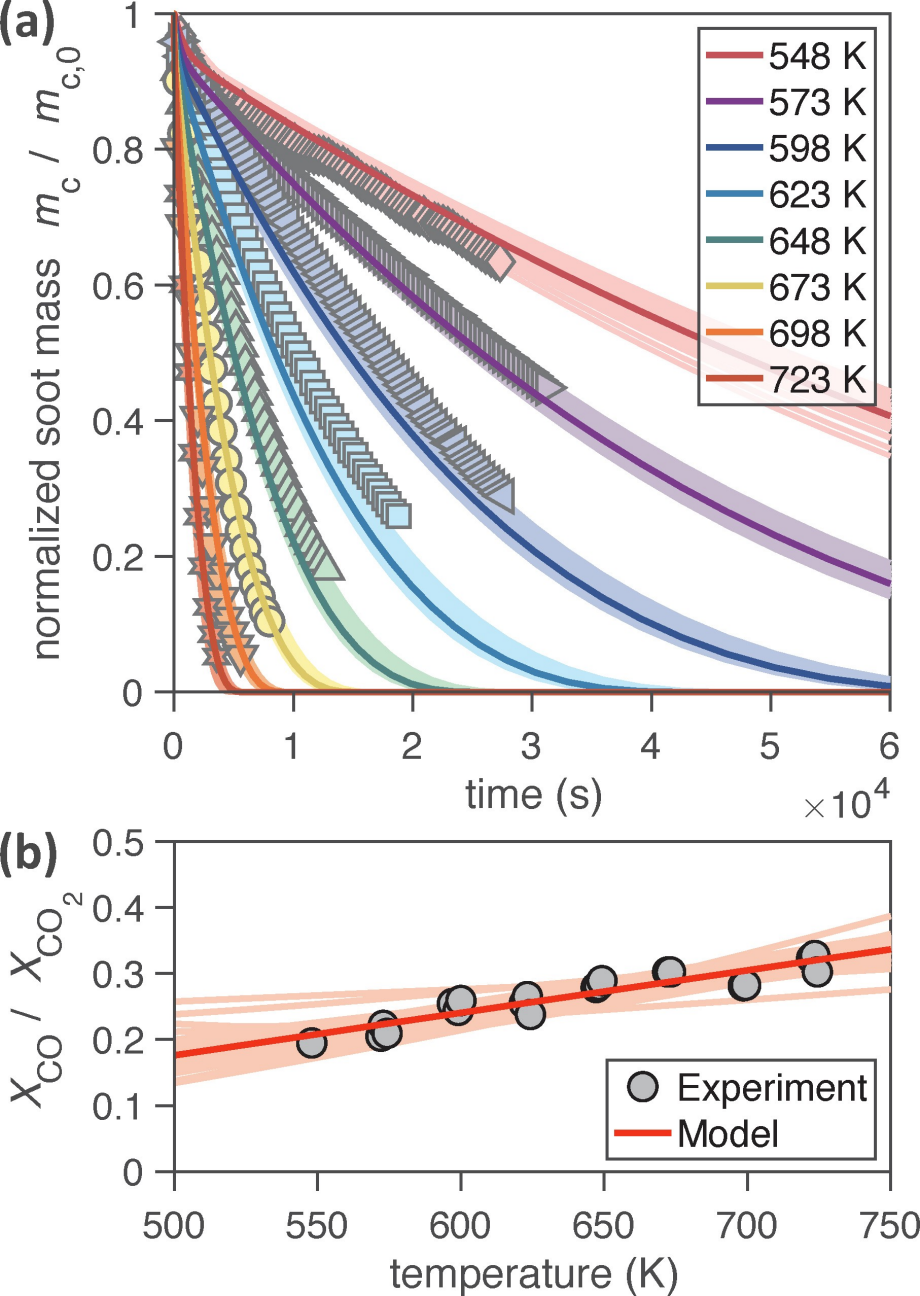
Carbon nanoparticle oxidation and gasification by NO_2_ (150 ppm), O_2_ (10 %), and H_2_O (3 %). (a) Decay of normalized soot mass over time for eight experiments at *T*=548–723 K and (b) the ratio of the mole fractions (*X*) of CO and CO_2_ in the reactor outflow as a function of temperature. Experimental data (markers)^[28]^ is compared with KM‐GAP‐CARBON model output (lines). Thick solid lines represent the globally best‐fitting kinetic parameter set, while shadings indicate the variability within the fit ensemble (Tables S1 and S2).

Figure 3a shows measurement and model results for the mass‐based pseudo‐first‐order rate coefficient of carbon oxidation ($k_{m}=\left(-dm_{C}/m_{C}\right)/dt$ ) plotted against reaction progress ($\xi =1-m_{C}/m_{C,0}$ ) and normalized by the rate coefficient observed at 50 % reaction progress (*k*_*m*,0.5_).^[28]^ The characteristic behavior of the system, an initial decrease and subsequent increase of *k_m_*, is well captured by our model, in the normalized plot (Figure 3a) as well as in individual plots of the experimental data recorded at different temperatures (Figure S1). The near‐linear increase of *k_m_* in the range of ξ≈0.1-0.9 and the steep increase at ξ>0.9 can be attributed to the increasing surface‐to‐volume ratio of the oxidized particles (shrinking core effect).

**Figure 3:**
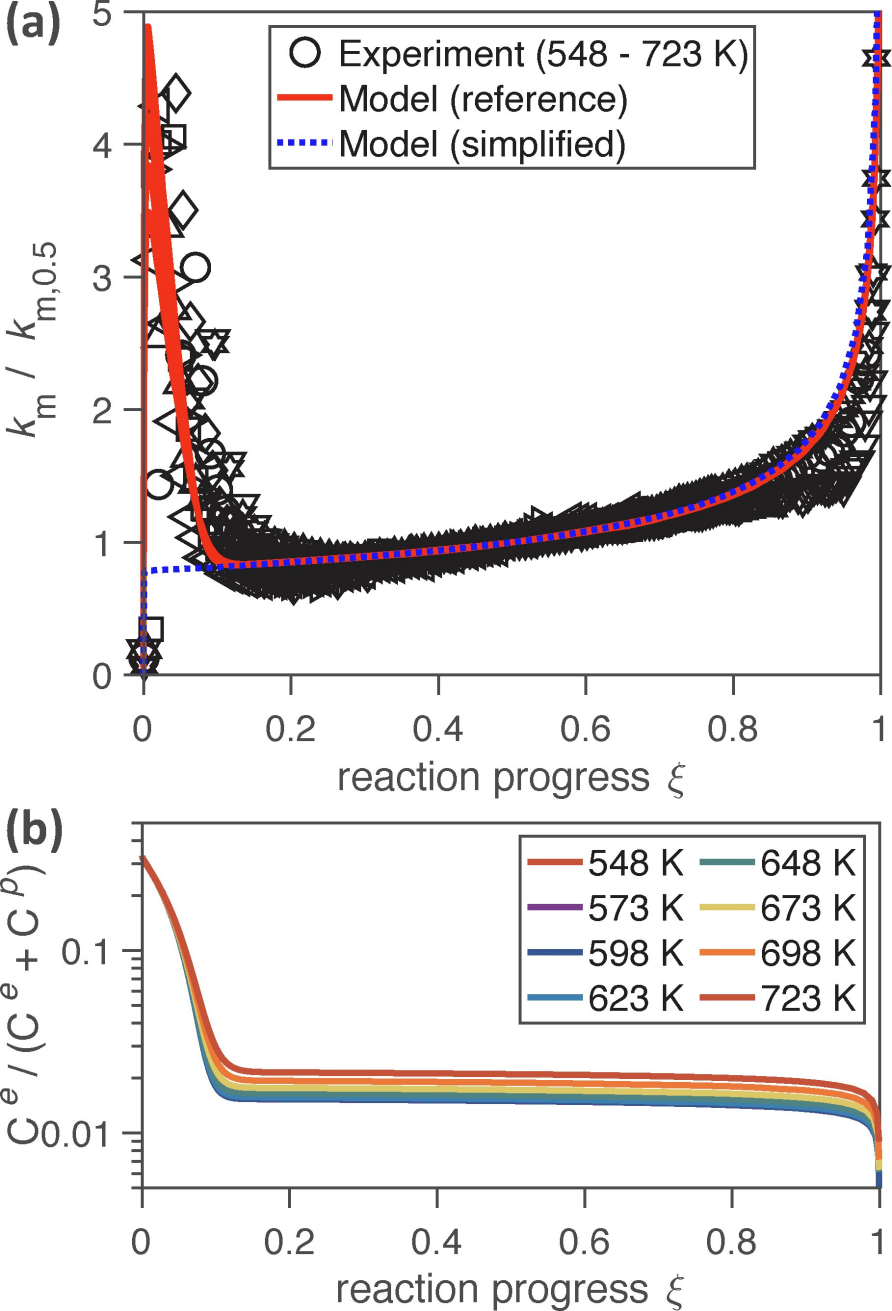
Changing oxidation rates and differential reactivity of carbon nanoparticles. (a) Mass‐based, first‐order reaction rate coefficient *k_m_* normalized by *k*_*m*,0.5_ and plotted against reaction progress *ξ*. Experimental data are represented by black open markers with different shapes for different temperatures (*T*=548–723 K; 150 ppm NO_2_, 10 % O_2_, 3 % H_2_O).^[28]^ Model results are represented by solid red lines for the full reference mechanism (548–723 K) and a dotted blue line for a simplified reaction mechanism without differential reactivity (623 K, mechanism C in Section 3 of the Supporting Information). (b) Mole fraction of edge‐like carbon atoms, C^*e*^, with higher reactivity than basal plane‐like carbon atoms, C^*p*^.

The pronounced decrease of *k_m_* in the early stage of oxidation (ξ<0.1 ) can be explained by changes in the chemical reactivity of the carbon nanoparticles and requires a chemical mechanism that accounts for the differential reactivity of different types of carbon atoms (C^*e*^, C^*p*^) as included in the KM‐GAP‐CARBON reference mechanism (solid red lines). The behavior cannot be described by a simplified mechanism that does not differentiate between more and less reactive carbon atoms (dotted blue line, mechanism C in Section 3 of the SI). The simplified mechanism yields similar results as traditional shrinking core model approaches, in which *k_m_* for ideal spheres is proportional to ${\left(1-\xi \right)}^{-1/3}$ .[^10^, ^28^] As illustrated in Figure 3b, preferential oxidation leads to depletion of the more reactive edge‐like carbon atoms (C^*e*^) at the particle surface. Accumulation of less‐reactive basal plane‐like carbons (C^*p*^) causes an effective shielding of C^*e*^ atoms in the particle bulk, and leads to a reduced overall oxidation rate that is governed by the reactivity of C^*p*^ atoms that remain accessible to gaseous oxidants. At ξ≈0.1 , the carbon mole fractions at the particle surface reach steady‐state levels around ~2 % C^*e*^ and ~98 % C^*p*^, respectively, exhibiting only minor dependencies on reaction temperature (Figure 3b).

The model results are consistent with experimental observations of a depletion of “active sites” upon oxidation of graphite.^[49]^ The initial bulk and surface mole fraction of 34 % edge‐like carbon atoms obtained by global optimization with our model (ξ=0 , Figure 3b) is also consistent with the 37.5 % determined by X‐ray diffraction analysis of graphite,^[55]^ while the fraction can vary widely for other types of carbonaceous materials.^[50]^ The median ratio between the initial NO_2_ oxidation rate coefficients for C^*e*^ (R1a) and C^*p*^ (R1b) obtained by global model optimization increases from ~25 at 550 K to ~40 at 750 K (Table S4), which is also consistent with experimentally determined factors of 10–100 reported in the Ref. [56].

Preferential reaction of certain sites may lead to the formation of porous nano‐ or microstructures that may influence the kinetics of mass transport and chemical reaction at gas‐particle interfaces,[^51^, ^57^, ^58^, ^59^] but our model assumes spherical particle geometry and does not resolve pore structures. Hence, the numerical values obtained by global optimization of our model for rate coefficients and the C^*e*^ mole fraction are effective values implicitly accounting for potential effects of pore structures. As the observed reaction rates are not fast enough to be limited by gas diffusion, however, we would not expect a strong influence of porosity on reaction kinetics and parameters.

According to our model analysis, preferential oxidation of edge‐type carbon atoms C^*e*^ is mostly driven by the differences in the NO_2_+C reaction rate, while we find no significant differences for O_2_+C (Tables S1 and S2). This may reflect differences in the reactivity of the two oxidants, but it also might be due to a lack of time‐resolved model training data for the O_2_+C reaction system. The slopes of measurement data and model lines in Figure 2a are in good agreement, while some model lines exhibit small but systematic offsets. These offsets may be due to differences in the initial morphology or composition of the investigated soot samples, e.g. in the ratio of surface‐exposed C^*e*^ and C^*p*^, which in turn might be due to temperature‐induced sintering effects.^[45]^

Figure 4a shows an Arrhenius plot for the pseudo‐first order rate coefficients observed at 50 % reaction progress, *k*_*m*,0.5_. The reaction system with NO_2_ and O_2_ (red symbols) exhibits a non‐linear dependence on inverse temperature, which indicates that the overall rate of carbon oxidation as represented by *k*_*m*,0.5_ is governed by more than a single reaction pathway. As illustrated by the tangents (dotted grey lines), the observable activation energy (*E*_a_) increases from only 60±5 kJ mol^−1^ at 549 K to 97±8 kJ mol^−1^ at 723 K (mean values and standard deviations across the fit ensemble obtained by global optimization).

**Figure 4:**
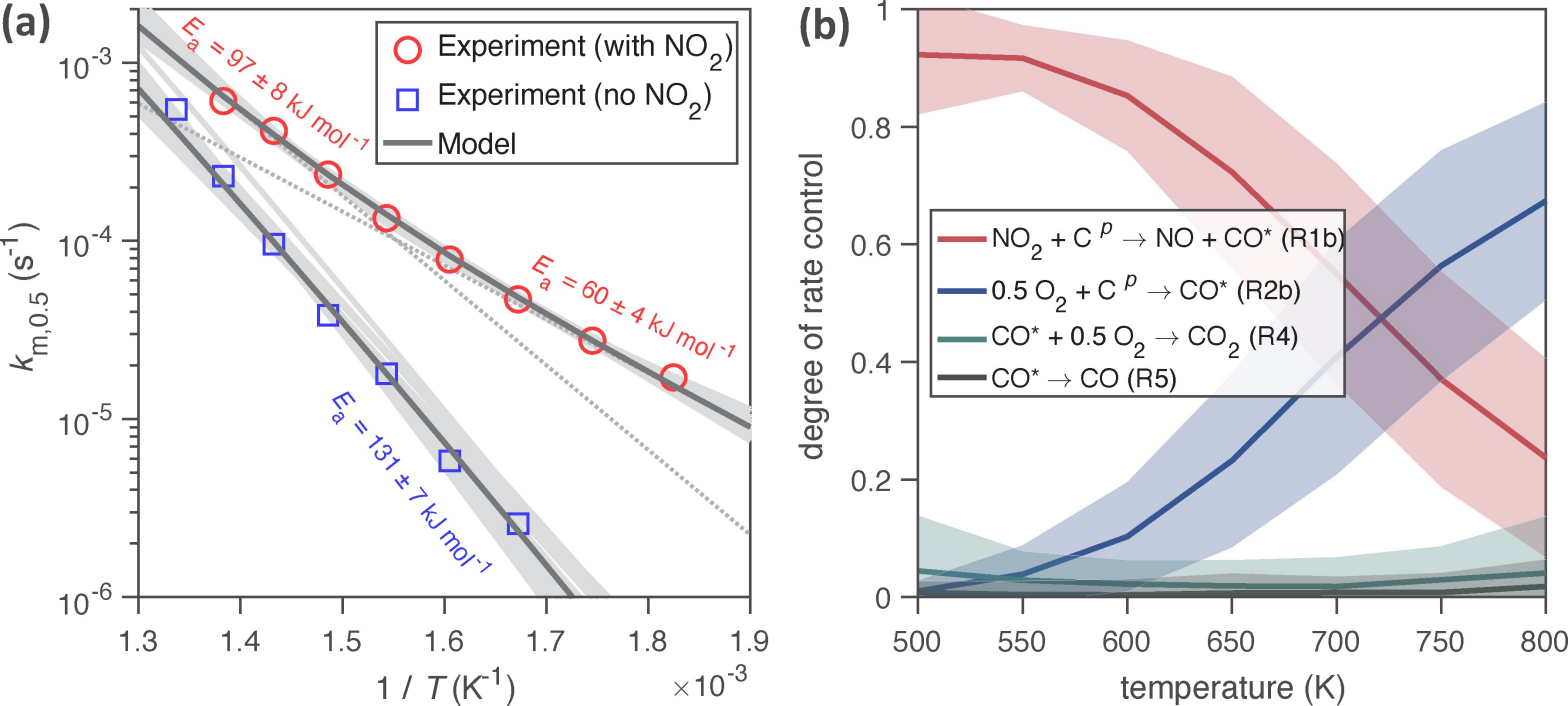
(a) Arrhenius plot for the mass‐based pseudo first‐order rate coefficient, *k*_*m*,0.5_, observed at 50 % reaction progress of carbon nanoparticle oxidation. *k*_*m*,0.5_ is shown as a function of inverse temperature for the reaction system with 150 ppm NO_2_, 10 % O_2_, and 3 % water vapor (red circles, upper curve) and for the same system without NO_2_ (blue squares, lower curve). The solid lines represent globally optimized model results (KM‐GAP‐CARBON with reference chemical mechanism), where the black line represents the globally best‐fitting kinetic parameter set and shadings indicate the variability within the fit ensemble. Activation energies (*E*_a_) are derived from slopes of tangents (dotted grey lines) for the reaction system with NO_2_ and directly from the slope of the model outputs (solid line) for the system without NO_2_. (b) Analysis of the degree of rate control (DRC)[^60^, ^61^] for the global rate of soot loss as a function of temperature. Solid lines indicate the mean of the fit ensemble, while shadings indicate two standard deviations around the mean. Displayed are the reactions with the highest DRC.

Figure 4a also depicts *k*_*m*,0.5_ from separate experiments in the absence of NO_2_ and thus O_2_ as main oxidant (blue symbols and lower curve). We find this system to follow Arrhenius behavior and determine an activation energy of 131±7 kJ mol^−1^. This finding agrees well with Yezerets et al.,^[62]^ who determined 137 kJ mol^−1^ for the reaction of diesel soot with O_2_ between 673 and 823 K. Furthermore, the value is similar to Lee et al.,^[63]^ who reported 155 kJ mol^−1^ between 789 and 899 K, and Leistner et al.,^[41]^ who reported individual *E*_a_ of 147 and 164 kJ mol^−1^ for the reactions to CO and CO_2_, respectively.

As illustrated in Figure 4b, a model analysis of the degree of rate control (DRC)[^60^, ^61^] indicates that the overall rate of carbon oxidation is largely controlled by the reaction of NO_2_ with carbon (R1b) at temperatures below ~700 K, whereas O_2_ (R2b) becomes the dominant oxidant above ~750 K. Thus, the change in *E*_a_ as a function of the temperature in Figure 4a can be attributed to a change of chemical regimes and dominant reaction pathways with NO_2_ being predominant at low temperatures, while at higher temperatures, the observed *E*_a_ increases due to the influence of oxidation with O_2_. These model results are consistent with the experimental results that show a convergence at high *T* between the two reaction systems in Figure 4a. Furthermore, the low‐temperature *E*_a_ value of 60±5 kJ mol^−1^ is close to *E*_a_ values previously determined for the gasification of different types of soot by NO_2_: 50 kJ mol^−1^ for graphitized soot;^[64]^ 47.1 kJ mol^−1^ for flame soot;^[65]^ and 46–59 kJ mol^−1^ for carbon black.^[66]^

The observable activation energy values derived from the slope of the measured overall oxidation rates plotted in Figure 4a can be related to the *E*_a_ values obtained upon global optimization of our model for the initial steps of carbon oxidation by NO_2_ (R1) and O_2_ (R2) (Table S2). Note, however, that desorption energies have to be taken into account for this comparison, because the observable overall *E*_a_ value equals the difference between the standard enthalpy of the transient state of the rate‐limiting step and the standard enthalpy of the initial states of the reagent.^[67]^ Thus, the observable activation energy value equals the activation energy of the rate‐limiting step (R1) minus the desorption energy of the gas‐phase reagent (NO_2_) as illustrated and detailed in the following energy profiles for the overall reaction. Desorption energies of reaction products did not affect the model outcome.

Figure 5 shows energy profiles for the overall process of carbon oxidation and gasification which includes multiple steps as described in our model (KM‐GAP‐CARBON with reference mechanism): adsorption of reactants to the surface, reaction of carbon atoms at the surface with NO_2_ (panels a, b) or O_2_ (panel c) forming a surface‐bound ROI or activated complex CO*, decomposition of CO*, and desorption of physisorbed reaction products. The decomposition of CO* may occur as a first‐order reaction (panel a) or proceed through a second‐order reaction with NO_2_ (panel b) or O_2_ (panel c). Note that not all reactions in Figure 5 can be regarded as elementary steps, and may rather be effective reactions where the activation energies represent the rate‐limiting step. For example, the reaction of the carbon surface with O_2_ may evolve through addition to the carbon surface (COO* intermediate), followed by decomposition into CO*, or via dissociation into chemisorbed oxygen atoms.^[69]^

**Figure 5:**
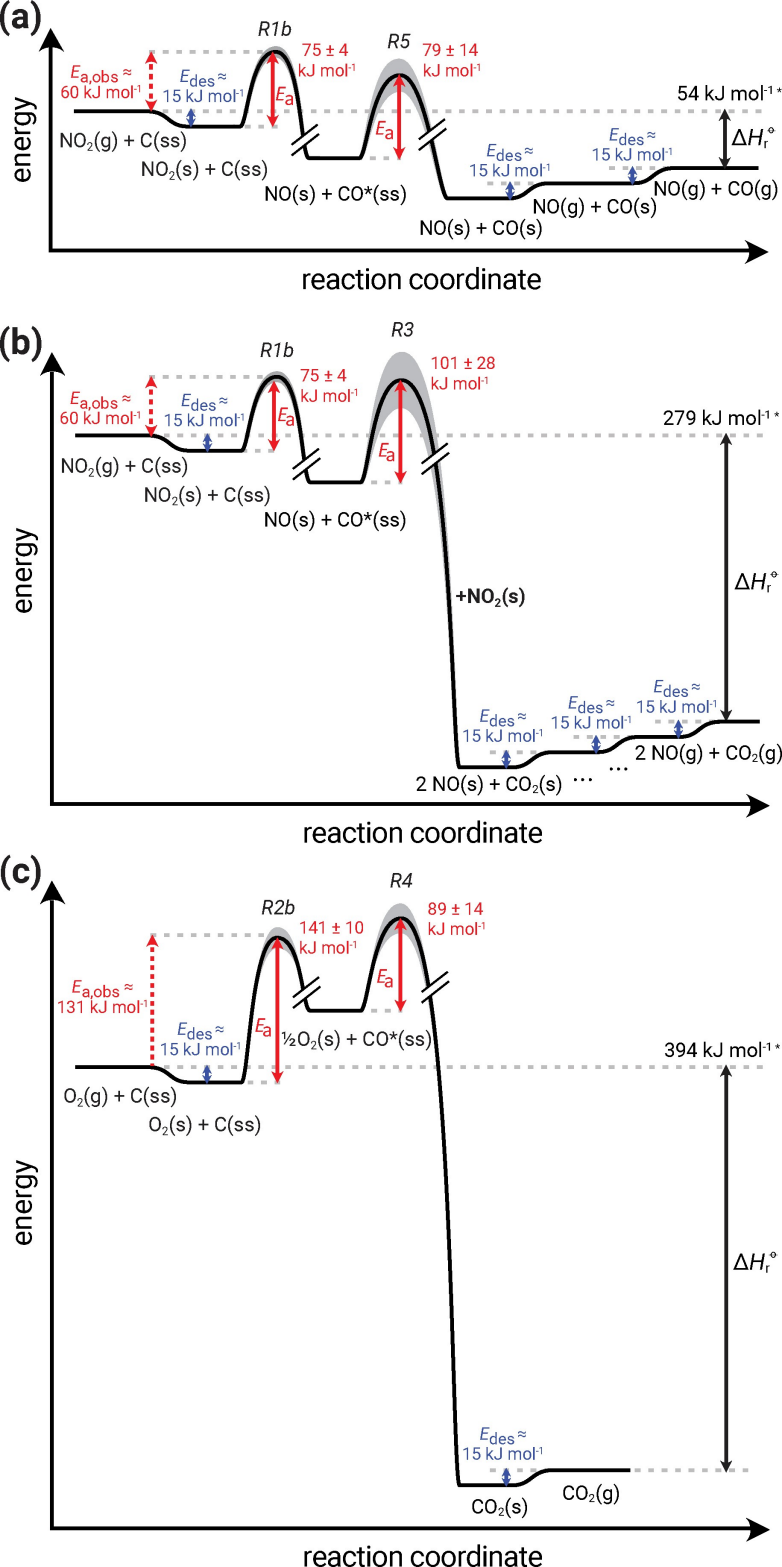
Energy profiles for the gasification of carbon nanoparticles with NO_2_ and O_2_ in KM‐GAP‐CARBON. (a) Reaction pathway involving unimolecular ROI‐decomposition (R1b, R5), (b) reaction pathway involving bimolecular ROI decomposition with NO_2_ (R1b, R3), (c) reaction pathway involving bimolecular ROI decomposition with O_2_ (R2b, R4). The multi‐step process is broken down into physical adsorption/desorption processes and chemical reactions. For physisorption, a temperature‐independent desorption energy (*E*_des_, blue arrows) of 15 kJ mol^−1^ is used.^[68]^ Activation barriers for the chemical reactions (*E*_a_, red arrows) stem from global optimization and parameter uncertainty (grey shadings) corresponds to the standard deviation within the fit ensemble.

The reaction enthalpies of the sum reactions ($\Delta H_{r}^{-o}$ , black arrow) are markedly different between the reactions pathways, with the pathways including the second‐order reactions of NO_2_ and O_2_ with CO* being strongly exothermic due to the stability of the formed CO_2_. $\Delta H_{r}^{-o}$ are derived from standard formation enthalpies at 298.15 K, calculated using polynomials tabulated in the NASA Glenn thermodynamic database,^[70]^ and hence denoted with an asterisk. The temperature dependencies of the $\Delta H_{r}^{-o}$ are found to be minor (Figure S2). The activation enthalpies for the chemical reactions (*E*_a_) are given as averages±one standard deviation from the globally optimized fit ensemble. The value of the observable activation energy of the global reaction identified from the Arrhenius plot ($E_{a,\mathrm{obs}}$ is identified here as the energy between gaseous oxidants and the first transient state).

This finding, together with our earlier analysis of the degree of rate control in the kinetic model (Figure 4a), reveals that the formation of the surface‐active complex constitutes the bottleneck of the global reaction under the reaction conditions investigated in this study. This is markedly different to the high‐temperature gasification of graphite or charcoal with CO_2_ (Boudouard reaction) in which the first reaction step is reversible and the second reaction step, desorption of CO* (R5), is usually identified as rate‐limiting.^[35]^ The change of dominant reaction pathways reflects important differences in the intrinsic properties (reactivity of oxidants) and extrinsic conditions (temperatures and concentration levels) of the two reaction systems. We suggest that this may be due to the higher reactivity of CO* with NO_2_ and O_2_ compared to CO_2_, leading to lower steady‐state concentrations of ROI at the particle surface and a smaller contribution of unimolecular decomposition. The lower reactivity of CO_2_ is intuitive given the high chemical stability of the compound and due to the large amount of energy required to break the C=O double bond. At the high temperatures required to oxidize elemental carbon by CO_2_, the unimolecular decomposition of CO* (R5) may proceed faster than the bimolecular decomposition with a second equivalent of oxidant (CO*+CO_2_), and become the rate‐determining step. This may contribute to the propensity of the C+CO_2_ system to exhibit saturation effects (insensitivity to increasing oxidant concentration), and significant back reaction from CO* to CO_2_, as discussed by Ergun and others.[^35^, ^36^, ^71^] For the NO_2_+C system and under the given reaction conditions, we do not find evidence for a noticeable influence of back reactions or reaction products.

Oxidant concentrations may also play role in the competition of formation and desorption of CO*. When extrapolating the model to higher NO_2_ concentrations (Figure S3), we see the beginning of a transition to limitation by desorption of CO* through reactions R4 and R5, especially at lower temperatures. Large error bars, i.e., a large variability within the fit ensemble, however, indicate a larger uncertainty for this finding. In future investigations, we intend to use the KM‐GAP‐CARBON model as guide to identify optimal experimental conditions using a kinetic compass method that utilizes machine learning and neural network surrogate modelling.[^27^, ^72^]

The oxidation of carbon by water vapor is known to require higher temperatures than probed in this study,^[73]^ but minor accelerating effects of H_2_O on the gasification of carbon by NO_2_ and O_2_ have been reported.^[74]^ We performed global optimization on an expanded chemical mechanism including water vapor (mechanism B in Supporting Information Section 3), found no substantial differences, and did not include water reactions in our reference mechanism, because these reactions are not well constrained and have little impact under the given conditions (Figure S9).

Overall, we show that the concentration and temperature dependence of carbon nanoparticle oxidation by NO_2_ and O_2_ can be described by a chemical mechanism that involves the formation of a surface‐bound reactive oxygen intermediate (ROI) in the form of an activated carbon complex CO*. We find that the rate‐limiting step of carbon oxidation under the given conditions is the formation rather than the decomposition and desorption of the ROI.

According to the experimental data and the energy profiles derived for alternative reaction pathways, the ROI formation is dominated by NO_2_ with an activation energy of *E_a_* =75±4 kJ mol^−1^ at lower temperatures (<600 K). At higher temperatures, we observe a transition to a different chemical regime where O_2_ substantially contributes to ROI formation with an activation energy of *E_a_* =141±10 kJ mol^−1^). Analyzing the degree of reaction control by different oxidants and reaction pathways, we find that the decomposition of CO* proceeds predominantly via bimolecular reaction with a second equivalent of oxidant. This is markedly different to the high‐temperature carbon oxidation by CO_2_ (Boudouard reaction), where CO* is assumed to decompose mainly through a unimolecular, first‐order reaction.^[75]^

In accordance with related earlier studies, we find that edge‐like carbon atoms (C^*e*^) react by a factor of 25–40 faster than basal plane‐like atoms (C^*p*^). Upon oxidant exposure, C^*e*^ atoms are thus depleted at the surface (~2 %) relative to the bulk material (~34 %), which leads to an effective passivation, reduces the observed global reaction rate, and affects the temporal evolution of carbon mass loss. Additional experiments are needed to further constrain the influence of water vapor and the reactivity of different oxidants, including NO_2_, O_2_, H_2_O, and CO_2_, with C^*e*^ and C^*p*^ atoms under any kind of reaction conditions. Such reactions may also contribute to the development of new technologies in carbon capture and synthetic fuel production.[^76^, ^77^]

## Conflict of Interests

The authors declare no competing interests.

## Supporting information

As a service to our authors and readers, this journal provides supporting information supplied by the authors. Such materials are peer reviewed and may be re‐organized for online delivery, but are not copy‐edited or typeset. Technical support issues arising from supporting information (other than missing files) should be addressed to the authors.

Supporting Information

## Data Availability

Experimental data was derived from published resources. All model parameters obtained in this study are provided in the Supporting Information.
