# Supplementary material for: Carbon Nanoparticle Oxidation by NO2 and O2: Chemical Kinetics and Reaction Pathways
Source: Angew Chem Int Ed Engl. 2024 Nov 26;63(52):e202413325. doi: 10.1002/anie.202413325 (PMC11656147; doi:10.1002/anie.202413325)
Supplement: Supplementary file 1 — Supporting Information [file ANIE-63-e202413325-s001.pdf]

Angewandte Chemie
Eine Zeitschrift der Gesellschaft Deutscher Chemiker

Supporting Information

Carbon Nanoparticle Oxidation by NO_2 and O_2: Chemical Kinetics and Reaction Pathways

T. Berkemeier, U. Pöschl*

Supporting Information: Carbon Nanoparticle Oxidation by NO_2 and O_2: Chemical Kinetics and Reaction Pathways

Thomas Berkemeier[1] and Ulrich Pöschl[1]

[1]Multiphase Chemistry Department, Max Planck Institute for Chemistry, Hahn-Meitner-Weg 1, 55128 Mainz, Germany

Correspondence: Thomas Berkemeier (t.berkemeier@mpic.de)

S1 KM-GAP-CARBON model

As illustrated in Fig. S4, KM-GAP-CARBON comprises multiple model compartments and layers, respectively: gas phase (g), sorption layer (s), quasi-static surface layer (ss), and n bulk layers (b). For this study, we assume a homogeneous bulk of soot particles and use a single bulk layer (n=1). The sorption and quasi-static surface layer are monolayers consisting of physisorbed and chemisorbed chemical species, respectively. The condensed phase can accumulate or lose mass through adsorption, desorption, and surface-bulk exchange. Desorption lifetimes are calculated in a temperature-dependent manner from desorption energies following Knopf et al. (2024). The bulk volume can shrink or grow in response to mass transport and chemical reactions. KM-GAP-CARBON can deal with spherical and planar geometries (thin films) and accounts for the following processes: gas-phase diffusion, gas–surface transport (reversible adsorption), surface–bulk transport, surface and bulk reactions, and bulk diffusion. The bulk volume shrinks and grows in response to mass transport and chemical reactions.

The rate equations describing the production, loss, and transport (mass balance) of each chemical species in each model compartment are listed in Section S5. The coupled ordinary differential equations are solved in Matlab software using a stiff differential equation solver (ode23tb) which is an implementation of TR-BDF2, a numerical method using an implicit Runge-Kutta formula (trapezoidal rule) and a second-order backward differentiation formula. Kinetic model parameters are determined via global model optimization using a Monte-Carlo genetic algorithm for unbiased multi-parameter fitting to existing experimental data (Berkemeier et al., 2017), which is further described in Section S5. Instead of a single best kinetic parameter set, an ensemble of parameter sets (N=50) is obtained to represent model parametric uncertainty from the global optimization (Berkemeier et al., 2021). Hence, model parametric uncertainty is determined in a process related to approximate Bayesian computation (Krüger et al., 2024).

Soot in the flat bed reactor is assumed to consist of monodisperse primary particles with a diameter of 10 nm. These primary particles are treated in the model as individual, non-touching, perfect spheres, which implies an initial surface-to-volume ratio of 0.3 nm^2/nm^3. We further assume a primary particle density of 1.5 g cm^{-3}, which is a central value in the range typically reported in experimental observations (Ouf et al., 2019). An overview of the KM-GAP-CARBON model parameters that remained fixed during global optimization is given in Table S4. Gas diffusion effects are negligible at the high temperatures

and comparatively slow reaction rates in this study. Likewise, we find no significant effect of gas-phase chemistry (e.g. NO_2 + CO $\longrightarrow$ CO_2 + NO) on the composition of reactor outflow.

The activated surface complex CO^* resides in the quasi-static surface layer, but also occupies a site in the sorption layer. A molecule of CO^* leaving the quasi-static surface layer is replaced in the model by an underlying carbon atom from the first bulk layer through bulk–surface transport. The likeliness that a carbon is replaced by a C^e or C^p atom corresponds to their concentration in the bulk. C^e and C^p atoms are randomly distributed across the particle in the model. The chemical mechanism does not distinguish between CO^* formed from C^e and C^p atoms.

For simplicity and following earlier studies, we assume that the soot particles are composed of carbon only. In reality, soot may encompass C/H ratios of 1.4-20, depending on soot maturity, and may also contain oxygenated species (Alfè et al., 2009; Michelsen, 2017). While nascent soot contains a large fraction of PAH, the aggregation, dehydrogenation and isomerization of PAH leads to fullerenes and consecutively to larger, graphitic carbon structures (Dobbins et al., 1998; Reilly et al., 2000; Mansurov, 2005; Sabbah et al., 2021).

We find no effect of subdividing the particle bulk into concentric diffusion layers (Shiraiwa et al., 2012). This test requires the use of an automated layer merging scheme that incorporates the shrinking first, outer bulk layer into the second bulk layer once the thickness of the outer layer falls below a monolayer of carbon atoms. This is accomplished by successively stopping integration of the ODE and re-starting the model with the new layer count (Berkemeier et al., 2020).

Using the GRI-Mech 3.0 chemical mechanism (Smith et al., retrieved 2024), we find that gas-phase oxidation of CO plays only a minor role in the formation of CO_2.

S2 Experimental data

Experiments were performed by Messerer et al. (2006) in a flat bed reactor for temperatures between 548 and 723 K in steps of 25 K. The flat bed reactor (Messerer et al., 2004) with a volume of 21 cm^3 was loaded with a soot mass of 7.5 mg. The reactor feed gas flow was 5 NL min^{-1} with inflow concentrations of 0-150 ppm NO_2, 0-45 ppm NO, 0.3-10 % O_2, and 0-8.2 % water vapor. Experimental conditions are detailed in Supplementary Table S3.

S3 Chemical reactions mechanisms

The reference chemical reaction mechanism employed in this study involves the formation of a single reactive oxygen intermediate (ROI), CO^*, at the soot surface and provides a representation of soot nanostructure through the distinction of edge-like (C^e) and basal plane-like carbon atoms (C^p).

Mechanism A - reference mechanism

The reference mechanism in this study, describes a single reactive oxygen intermediate (ROI) and two types of carbon sites (edge, basal plane) to account for soot nanostructure, is given in Eqs. R1a-R5, and is utilized for most calculations in the main

text of this study. Note that, in reality, multiple surface functional groups are known to exist on the soot surface, which could be described in a reaction mechanism including multiple ROI (multi-ROI mechanism). In this study, we opted for a single-ROI mechanism as it proved sufficient to describe most of the features in the experimental data. The number of chemical species and reactions were kept as low possible and as high as necessary to describe the essential features of the experimental data.

$$\mathrm{NO_2\,(s) + C^{\mathit{e}}\,(ss) \longrightarrow NO\,(s) + CO^{*}\,(ss)} \tag{R1a}$$

$$\mathrm{NO_2\,(s) + C^{\mathit{p}}\,(ss) \longrightarrow NO\,(s) + CO^{*}\,(ss)} \tag{R1b}$$

$$\frac{1}{2}\,\mathrm{O_2\,(s) + C^{\mathit{e}}\,(ss) \longrightarrow CO^{*}\,(ss)} \tag{R2a}$$

$$\frac{1}{2}\,\mathrm{O_2\,(s) + C^{\mathit{p}}\,(ss) \longrightarrow CO^{*}\,(ss)} \tag{R2b}$$

$$\mathrm{CO^{*}\,(ss) + NO_2\,(s) \longrightarrow NO\,(s) + CO_2\,(s)} \tag{R3}$$

$$\mathrm{CO^{*}\,(ss)} + \frac{1}{2}\,\mathrm{O_2\,(s) \longrightarrow CO_2\,(s)} \tag{R4}$$

$$\mathrm{CO^{*}\,(ss) \longrightarrow CO\,(s)} \tag{R5}$$

Through global optimization (Sect. S4), we obtain a fit ensemble with $N = 50$ fits and a mean root mean square error R (Eq. 1) of 4.51, with a standard deviation of 0.39 and a best value of 3.95. Our reference chemical mechanism goes beyond the unified gasification mechanism of Chen et al. (1993) in that it allows the formation of CO_2, which is a major reaction product in the oxidation of carbon with NO_2 (Stanmore et al., 2001) and O_2 (Li and Brown, 2001). This two-step oxidation where a second equivalent of oxidant can accelerate the desorption of the surface complex has been proposed based on experimental observations (e.g. Muckenhuber and Grothe, 2006).

Mechanism B - expanded reference mechanism

As first alternative to the reference mechanism, we present a mechanism in which the same chemistry that is observed for NO_2 and O_2 is expanded to water vapor, H_2O.

$$NO_2(s) + C^e(ss) \longrightarrow NO(s) + CO^*(ss) \tag{R1a}$$

$$NO_2(s) + C^p(ss) \longrightarrow NO(s) + CO^*(ss) \tag{R1b}$$

$$\frac{1}{2} O_2(s) + C^e(ss) \longrightarrow CO^*(ss) \tag{R2a}$$

$$\frac{1}{2} O_2(s) + C^p(ss) \longrightarrow CO^*(ss) \tag{R2b}$$

$$CO^*(ss) + NO_2(s) \longrightarrow NO(s) + CO_2(s) \tag{R3}$$

$$CO^*(ss) + \frac{1}{2} O_2(s) \longrightarrow CO_2(s) \tag{R4}$$

$$CO^*(ss) \longrightarrow CO(s) \tag{R5}$$

$$H_2O(s) + C^e(ss) \longrightarrow H_2(s) + CO^*(ss) \tag{R6a}$$

$$H_2O(s) + C^p(ss) \longrightarrow H_2(s) + CO^*(ss) \tag{R6b}$$

$$CO^*(ss) + H_2O(s) \longrightarrow H_2(s) + CO_2(s) \tag{R7}$$

We provide an $N = 20$ fit ensemble for the 20 kinetic parameters (two for each chemical reaction) of mechanism B. The kinetic model parameters determined from repeated global optimization are tabulated in Tables S6 and S7. Model results are shown in Figs. S9 and S10. Through global optimization (Sect. S4), we obtain a fit ensemble with $N = 20$ fits and a mean root mean square error R (Eq. 1) of 2.92, a standard deviation of 0.08 and a best value of 2.74.

Mechanism C - no distinction of carbon atoms

As another alternative to the reference mechanism, we present a reduced reaction mechanism to highlight the necessity of the representation of soot nanostructure to accurately describe the experimental data. Removal of the distinction of different carbon atoms yields the mechanism in Eqs. R8-R12. Reactions R10-12 are equivalent to reactions R3-R5, respectively.

$$NO_2(s) + C(ss) \longrightarrow NO(s) + CO^*(ss) \tag{R8}$$

$$\frac{1}{2} O_2(s) + C(ss) \longrightarrow CO^*(ss) \tag{R9}$$

$$CO^*(ss) + NO_2(s) \longrightarrow NO(s) + CO_2(s) \tag{R10}$$

$$CO^*(ss) + \frac{1}{2} O_2(s) \longrightarrow CO_2(s) \tag{R11}$$

$$CO^*(ss) \longrightarrow CO(s) \tag{R12}$$

Fig. S5a shows the KM-GAP-CARBON model output using the single-ROI mechanism without distinction of carbon atoms (mechanism C) after optimization of its 10 kinetic parameters (two for each chemical reaction) to the experimental data. The soot mass loss is overall captured, however, model-experiment correlation is worse than for the reference mechanism shown in Figs. 2 and 3 in the main text. Strikingly, the initial fast loss of soot is absent with this reaction mechanism (Fig. 3b), which

leads to an underestimation of the initial decay rate. The mass-based pseudo first-order rate coefficient at 50 % reaction progress ($k_{\mathrm{m,0.5}}$), however, is rather well-captured. Fig. S6 shows that the dependence of logarithmic $k_{\mathrm{m,0.5}}$ on inverse temperature is more linear with mechanism C than with the reference mechanism, which reduces model-experiment correlation slightly.

Mechanism D - concerted

Even further reduction of the reaction mechanism can be achieved through removal of the ROI, as shown in Eqs. R13-R15. We term this the concerted mechanism.

$$\mathrm{NO_2\,(s) + C\,(ss) \longrightarrow NO\,(s) + CO\,(s)} \tag{R13}$$

$$\mathrm{\frac{1}{2}\,O_2\,(s) + C\,(ss) \longrightarrow CO\,(s)} \tag{R14}$$

$$\mathrm{H_2O\,(s) + C\,(ss) \longrightarrow CO\,(s) + H_2\,(s)} \tag{R15}$$

Fig. S5b shows the KM-GAP-CARBON model output using the concerted mechanism D after optimization of its six kinetic parameters (two for each chemical reaction) to the experimental data. The soot mass loss is overall captured and model-experiment correlation is comparable to mechanism C (Fig. S5a, Fig. S6). The main limitation of the concerted mechanism D compared to mechanism C is that it only yields CO as reaction product and hence cannot describe the ratio of CO and CO_2 in the experimental data.

Mechanism E - CO* and COO* intermediates

The formation of CO_2 may involve a transformation of the initially formed ROI (CO^*) into a more oxidized ROI (COO^*). Accordingly, we tested an extended version of our mechanism explicitly resolving the formation of a COO^* intermediate, which is similar to the unified reaction mechanism presented for the CO_2 + soot reaction system in Chen et al. (1993). The mechanism presented here assumes similar reactivity for O_2, H_2O, and NO_2, and also tracks the type of carbon site for both, the unreacted carbons and the ROI (not spelled out in Eqs. R18-R26 for simplicity). Usage of the multi-ROI mechanism E in KM-GAP-CARBON did not lead to improvement in model-experiment correlation. Reactions R16-R19 are equivalent to reactions R1, R2, R6 and R5, respectively.

$$\mathrm{NO_2\,(s) + C\,(ss) \longrightarrow NO\,(s) + CO^*\,(ss)} \tag{R16}$$

$$\mathrm{\frac{1}{2}\,O_2\,(s) + C\,(ss) \longrightarrow CO^*\,(ss)} \tag{R17}$$

$$\mathrm{H_2O\,(s) + C\,(ss) \longrightarrow CO^*\,(ss) + H_2\,(s)} \tag{R18}$$

$$\mathrm{CO^*\,(ss) \longrightarrow CO\,(s)} \tag{R19}$$

$$\mathrm{CO^*\,(ss) + NO_2\,(s) \longrightarrow NO\,(s) + COO^*\,(ss)} \tag{R20}$$

$$\mathrm{CO^*\,(ss) + \frac{1}{2}\,O_2\,(s) \longrightarrow COO^*\,(ss)} \tag{R21}$$

$$\mathrm{CO^*\,(ss) + H_2O\,(s) \longrightarrow COO^*\,(ss) + H_2\,(s)} \tag{R22}$$

$$\mathrm{COO^*\,(ss) \longrightarrow CO_2\,(s)} \tag{R23}$$

$$\mathrm{COO^*\,(ss) + C\,(ss) \longrightarrow CO^*\,(ss) + CO\,(s)} \tag{R24}$$

With this mechanism, we tested a hypothesis that a good model-experiment correlation could also be obtained with a set of kinetic parameters that lead to overall reaction progress being limited by desorption or unimolecular reaction of the ROI, instead of the initial oxidation of C with NO_2, H_2O or O_2 (Fig. 4b in the main text). This is generally observed for high-temperature gasification of soot or char with CO_2 (Ergun, 1956). The hypothesis is tested by lowering the upper boundaries of the reaction rate coefficients of the reactions of NO_2, H_2O and O_2 with CO^* during global optimization. However, we find that the non-Arrhenius behavior of the NO_2/H_2O/O_2 + soot system cannot be explained with the multi-ROI mechanism E and this restriction. This is evidence that the kinetics of CO^* with NO_2 with soot in the range of 548-723 K must be different to those of CO_2 with soot at higher temperatures.

S4 Global optimization of kinetic model parameters

The kinetic parameters of the model KM-GAP-CARBON are constrained using the Monte Carlo Genetic Algorithm (MCGA) for unbiased global optimization of model input parameters by simultaneous fitting to multiple experimental data sets (Berkemeier et al., 2017). The MCGA is a two-step algorithm with the goal of minimizing model–experiment correlation. During the Monte Carlo stage of the optimization process, kinetic parameters are varied within the boundaries listed in Tab. S5. The algorithm assigns a fitness value R to each sampled parameter set in the form of a least-squares residual, quantifying the correlation to experimental data. Each of M data sets is normalized according to the number of data points and m_j its highest absolute data value.

$$R = \sum_{j=1}^{M} \sqrt{\frac{1}{m_j} \sum_{i=1}^{m_j} \left(\frac{Y_{\text{model}} - Y_{\text{experiment}}}{\max\left(\text{abs}(Y_{\text{experiment}})\right)} \right)^2} \tag{1}$$

During the genetic algorithm stage, the best model parameter sets are further improved using principles of natural evolution such as recombination, survival, and mutation. We fitted a total number of 21 input parameters to data of 37 laboratory experi-

ments with distinct reaction conditions. A full list of reaction conditions in the 37 flat bed reactor experiments of Messerer et al. (2006) we used in this study is given in Table S3. For experiments 1-8, not only a mass-based global rate coefficient $k_{\mathrm{m,0.5}}$ was reported by Messerer et al. (2006), but also the CO-to-CO_2 ratio in the exhaust gas as well as carbon mass and k_{m} as a function of reaction progress. All data were considered in the global optimization, which leads to experiments 1-8 being weighted four-times higher in model–experiment correlation than other experiments. The fitted kinetic parameters include the initial fraction of edge carbon atoms (f_{edge}), and two parameters each for Arrhenius parameterizations of ten temperature-dependent reaction rate coefficients (k) according to Eq. (2).

$$k(T) = k_{623\,\mathrm{K}} \cdot \exp\left(\frac{E_{\mathrm{A}}}{R}\left(\frac{1}{623\mathrm{K}} - \frac{1}{T}\right)\right) \tag{2}$$

The reaction rate coefficients k are temperature dependent and parameterized using the rate coefficient at 623 K ($k_{623\,\mathrm{K}}$), the activation energy of the reaction (E_{A}), and the gas constant R. This type of parameterization proved superior in the global optimization algorithm over more traditional Arrhenius equations using a pre-exponential factor A. A full overview of fitted parameters and their optimized parameter ranges are given in Tables S1 and S2. For each of N = 50 fit parameter sets, global optimization was performed using 5 328 000 model evaluations during the Monte Carlo stage and 6 660 000 model evaluations during the genetic algorithm step. Each optimization took between 20-24 hours on a computer cluster with 240 parallel CPUs. The fits obtained this way achieved a mean R of 4.45, with a standard deviation of 0.38 and a best value of 3.95. All fits were deemed sufficient within uncertainty of the experimental data.

S5 System of Ordinary Differential Equations

The differential equations that describe the mass or number balance of each molecule for surface and each bulk layers are given below. The coupled ordinary differential equations are solved in Matlab software using a stiff differential equation solver (ode23tb) which is an implementation of TR-BDF2, a numerical method using an implicit Runge-Kutta formula (trapezoidal rule) and a second-order backward differentiation formula. Symbols are defined and explained in Tab. S8.

S5.1 Gas phase

$$\frac{\mathrm{d}N_{\mathrm{NO2,g}}}{\mathrm{d}t} = \dot{V}_{\mathrm{flow}} \cdot [\mathrm{NO_2}]_{\mathrm{g,0}} - \dot{V}_{\mathrm{flow}}/V_{\mathrm{g}} \cdot N_{\mathrm{NO2,g}} - k_{\mathrm{a,NO2}} \cdot N_{\mathrm{NO2,g}} \cdot A_{\mathrm{s}}/V_{\mathrm{g}} + k_{\mathrm{d,NO2}} \cdot N_{\mathrm{NO2,s}} \tag{3}$$

$$\frac{\mathrm{d}N_{\mathrm{NO,g}}}{\mathrm{d}t} = \dot{V}_{\mathrm{flow}} \cdot [\mathrm{NO}]_{\mathrm{g,0}} - \dot{V}_{\mathrm{flow}}/V_{\mathrm{g}} \cdot N_{\mathrm{NO,g}} - k_{\mathrm{a,NO}} \cdot N_{\mathrm{NO,g}} \cdot A_{\mathrm{s}}/V_{\mathrm{g}} + k_{\mathrm{d,NO}} \cdot N_{\mathrm{NO,s}} \tag{4}$$

$$\frac{\mathrm{d}N_{\mathrm{CO,g}}}{\mathrm{d}t} = -\dot{V}_{\mathrm{flow}}/V_{\mathrm{g}} \cdot N_{\mathrm{CO,g}} - k_{\mathrm{a,CO}} \cdot N_{\mathrm{CO,g}} \cdot A_{\mathrm{s}}/V_{\mathrm{g}} + k_{\mathrm{d,CO}} \cdot N_{\mathrm{CO,s}} \tag{5}$$

$$\frac{\mathrm{d}N_{\mathrm{CO2,g}}}{\mathrm{d}t} = -\dot{V}_{\mathrm{flow}}/V_{\mathrm{g}} \cdot N_{\mathrm{CO2,g}} - k_{\mathrm{a,CO2}} \cdot N_{\mathrm{CO2,g}} \cdot A_{\mathrm{s}}/V_{\mathrm{g}} + k_{\mathrm{d,CO2}} \cdot N_{\mathrm{CO2,s}} \tag{6}$$

$$\frac{\mathrm{d}N_{\mathrm{O2,g}}}{\mathrm{d}t} = \dot{V}_{\mathrm{flow}} \cdot [\mathrm{O_2}]_{\mathrm{g,0}} - \dot{V}_{\mathrm{flow}}/V_{\mathrm{g}} \cdot N_{\mathrm{O2,g}} - k_{\mathrm{a,O2}} \cdot N_{\mathrm{O2,g}} \cdot A_{\mathrm{s}}/V_{\mathrm{g}} + k_{\mathrm{d,O2}} \cdot N_{\mathrm{O2,s}} \tag{7}$$

$$\frac{\mathrm{d}N_{\mathrm{H2O,g}}}{\mathrm{d}t} = \dot{V}_{\mathrm{flow}} \cdot [\mathrm{H_2O}]_{\mathrm{g,0}} - \dot{V}_{\mathrm{flow}}/V_{\mathrm{g}} \cdot N_{\mathrm{H2O,g}} - k_{\mathrm{a,H2O}} \cdot N_{\mathrm{H2O,g}} \cdot A_{\mathrm{s}}/V_{\mathrm{g}} + k_{\mathrm{d,H2O}} \cdot N_{\mathrm{H2O,s}} \tag{8}$$

S5.2 Sorption layer

$$\frac{\mathrm{d}N_{\mathrm{NO2,s}}}{\mathrm{d}t} = -k_{\mathrm{1a}} \cdot N_{\mathrm{NO2,s}} \cdot N_{\mathrm{C}^e\mathrm{,ss}}/A_{\mathrm{ss}} - k_{\mathrm{1b}} \cdot N_{\mathrm{NO2,s}} \cdot N_{\mathrm{C}^b\mathrm{,ss}}/A_{\mathrm{ss}} - k_4 \cdot N_{\mathrm{NO2,s}} \cdot N_{\mathrm{CO}^*\mathrm{,ss}}/A_{\mathrm{ss}} + k_{\mathrm{a,NO2}} \cdot N_{\mathrm{NO2,g}} \cdot A_{\mathrm{s}}/V_{\mathrm{g}} - k_{\mathrm{d,NO2}} \cdot N_{\mathrm{NO2,s}} \tag{9}$$

$$\frac{\mathrm{d}N_{\mathrm{NO,s}}}{\mathrm{d}t} = k_{\mathrm{1a}} \cdot N_{\mathrm{NO2,s}} \cdot N_{\mathrm{C}^e\mathrm{,ss}}/A_{\mathrm{ss}} + k_{\mathrm{1b}} \cdot N_{\mathrm{NO2,s}} \cdot N_{\mathrm{C}^b\mathrm{,ss}}/A_{\mathrm{ss}} + k_4 \cdot N_{\mathrm{NO2,s}} \cdot N_{\mathrm{CO}^*\mathrm{,ss}}/A_{\mathrm{ss}} + k_{\mathrm{a,NO}} \cdot N_{\mathrm{NO,g}} \cdot A_{\mathrm{s}}/V_{\mathrm{g}} - k_{\mathrm{d,NO}} \cdot N_{\mathrm{NO,s}} \tag{10}$$

$$\frac{\mathrm{d}N_{\mathrm{CO,s}}}{\mathrm{d}t} = k_7 \cdot N_{\mathrm{CO}^*\mathrm{,ss}} + k_{\mathrm{a,CO}} \cdot N_{\mathrm{CO,g}} \cdot A_{\mathrm{s}}/V_{\mathrm{g}} - k_{\mathrm{d,CO}} \cdot N_{\mathrm{CO,s}} \tag{11}$$

$$\frac{\mathrm{d}N_{\mathrm{CO2,s}}}{\mathrm{d}t} = k_4 \cdot N_{\mathrm{NO2,s}} \cdot N_{\mathrm{CO}^*\mathrm{,ss}}/A_{\mathrm{ss}} + k_5 \cdot N_{\mathrm{CO}^*\mathrm{,ss}} \cdot N_{\mathrm{O2,s}}/A_{\mathrm{ss}} + k_6 \cdot N_{\mathrm{CO}^*\mathrm{,ss}} \cdot N_{\mathrm{H2O,s}}/A_{\mathrm{ss}} + k_{\mathrm{a,CO2}} \cdot N_{\mathrm{CO2,g}} \cdot A_{\mathrm{s}}/V_{\mathrm{g}} - k_{\mathrm{d,CO2}} \cdot N_{\mathrm{CO2,s}} \tag{12}$$

$$\frac{\mathrm{d}N_{\mathrm{O2,s}}}{\mathrm{d}t} = -0.5 \cdot k_{\mathrm{2a}} \cdot N_{\mathrm{C}^e\mathrm{,ss}} \cdot N_{\mathrm{O2,s}}/A_{\mathrm{ss}} - 0.5 \cdot k_{\mathrm{2b}} \cdot N_{\mathrm{O2,s}}/A_{\mathrm{ss}} - 0.5 \cdot k_5 \cdot N_{\mathrm{CO}^*\mathrm{,ss}} \cdot N_{\mathrm{O2,s}}/A_{\mathrm{ss}} + k_{\mathrm{a,O2}} \cdot N_{\mathrm{O2,g}} \cdot A_{\mathrm{s}}/V_{\mathrm{g}} - k_{\mathrm{d,O2}} \cdot N_{\mathrm{O2,s}} \tag{13}$$

$$\frac{\mathrm{d}N_{\mathrm{H2O,s}}}{\mathrm{d}t} = -k_{\mathrm{3a}} \cdot N_{\mathrm{C}^e\mathrm{,ss}} \cdot N_{\mathrm{H2O,s}}/A_{\mathrm{ss}} - k_{\mathrm{3b}} \cdot N_{\mathrm{C}^b\mathrm{,ss}} \cdot N_{\mathrm{H2O,s}}/A_{\mathrm{ss}} \tag{14}$$

S5.3 Quasi-static surface layer

$$\frac{\mathrm{d}N_{\mathrm{CO}^*\mathrm{,ss}}}{\mathrm{d}t} = k_{\mathrm{1a}} \cdot N_{\mathrm{NO2,s}} \cdot N_{\mathrm{C}^e\mathrm{,ss}}/A_{\mathrm{ss}} + k_{\mathrm{1b}} \cdot N_{\mathrm{NO2,s}} \cdot N_{\mathrm{C}^b\mathrm{,ss}}/A_{\mathrm{ss}} - k_7 \cdot N_{\mathrm{CO}^*\mathrm{,ss}} - k_4 \cdot N_{\mathrm{NO2,s}} \cdot N_{\mathrm{CO}^*\mathrm{,ss}}/A_{\mathrm{ss}} + k_{\mathrm{2a}} \cdot N_{\mathrm{C}^e\mathrm{,ss}} \cdot N_{\mathrm{O2,s}}/A_{\mathrm{ss}} + k_{\mathrm{2b}} \cdot N_{\mathrm{C}^b\mathrm{,ss}} \cdot N_{\mathrm{O2,s}}/A_{\mathrm{ss}} - k_5 \cdot N_{\mathrm{CO}^*\mathrm{,ss}} \cdot N_{\mathrm{O2,s}}/A_{\mathrm{ss}} + k_{\mathrm{3a}} \cdot N_{\mathrm{C}^e\mathrm{,ss}} \cdot N_{\mathrm{H2O,s}}/A_{\mathrm{ss}} + k_{\mathrm{3b}} \cdot N_{\mathrm{C}^b\mathrm{,ss}} \cdot N_{\mathrm{H2O,s}}/A_{\mathrm{ss}} - k_6 \cdot N_{\mathrm{CO}^*\mathrm{,ss}} \cdot N_{\mathrm{H2O,s}}/A_{\mathrm{ss}} - k_6 \cdot N_{\mathrm{CO}^*\mathrm{,ss}} \cdot N_{\mathrm{H2O,s}}/A_{\mathrm{ss}} + k_{\mathrm{a,H2O}} \cdot N_{\mathrm{H2O,g}} \cdot A_{\mathrm{s}}/V_{\mathrm{g}} - k_{\mathrm{d,H2O}} \cdot N_{\mathrm{H2O,s}} \tag{15}$$

$$\frac{\mathrm{d}N_{\mathrm{C}^e\mathrm{,ss}}}{\mathrm{d}t} = -k_{\mathrm{1a}} \cdot N_{\mathrm{NO2,s}} \cdot N_{\mathrm{C}^e\mathrm{,ss}}/A_{\mathrm{ss}} - k_{\mathrm{2a}} \cdot N_{\mathrm{C}^e\mathrm{,ss}} \cdot N_{\mathrm{O2,s}}/A_{\mathrm{ss}} - k_{\mathrm{3a}} \cdot N_{\mathrm{C}^e\mathrm{,ss}} \cdot N_{\mathrm{H2O,s}}/A_{\mathrm{ss}} - k_{\mathrm{ssb,C}^e} \cdot N_{\mathrm{C}^e\mathrm{,ss}} + k_{\mathrm{bss,C}^e} \cdot N_{\mathrm{C}^e\mathrm{,b}} \cdot A_{\mathrm{b}}/V_{\mathrm{b}} \tag{16}$$

$$\frac{\mathrm{d}N_{\mathrm{C}^b\mathrm{,ss}}}{\mathrm{d}t} = -k_{\mathrm{1b}} \cdot N_{\mathrm{NO2,s}} \cdot N_{\mathrm{C}^b\mathrm{,ss}}/A_{\mathrm{ss}} - k_{\mathrm{2b}} \cdot N_{\mathrm{C}^b\mathrm{,ss}} \cdot N_{\mathrm{O2,s}}/A_{\mathrm{ss}} - k_{\mathrm{3b}} \cdot N_{\mathrm{C}^b\mathrm{,ss}} \cdot N_{\mathrm{H2O,s}}/A_{\mathrm{ss}} - k_{\mathrm{ssb,C}^b} \cdot N_{\mathrm{C}^b\mathrm{,ss}} + k_{\mathrm{bss,C}^b} \cdot N_{\mathrm{C}^b\mathrm{,b}} \cdot A_{\mathrm{b}}/V_{\mathrm{b}} \tag{17}$$

S5.4 Bulk layer

$$\frac{\mathrm{d}N_{\mathrm{C}^e\mathrm{,b}}}{\mathrm{d}t} = k_{\mathrm{ssb,C}^e} \cdot N_{\mathrm{C}^e\mathrm{,ss}} - k_{\mathrm{bss,C}^e} \cdot N_{\mathrm{C}^e\mathrm{,b}} \cdot A_{\mathrm{b}}/V_{\mathrm{b}} \tag{18}$$

$$\frac{\mathrm{d}N_{\mathrm{C}^b\mathrm{,b}}}{\mathrm{d}t} = k_{\mathrm{ssb,C}^b} \cdot N_{\mathrm{C}^b\mathrm{,ss}} - k_{\mathrm{bss,C}^b} \cdot N_{\mathrm{C}^b\mathrm{,b}} \cdot A_{\mathrm{b}}/V_{\mathrm{b}} \tag{19}$$

S5.5 Mass-transport coefficients

The first-order rate coefficients for mass transport include the adsorption rate coefficient k_a, the desorption rate coefficient k_d, the rate coefficient for surface–bulk transport k_ssb, and the rate coefficient for bulk–surface transport k_bss. For a molecule Z, the mass-transport coefficients are calculated as follows:

$$k_\mathrm{a,Z} = \alpha_\mathrm{s,0,Z} \cdot w_\mathrm{Z}/4 \cdot (1 - \theta_\mathrm{ss}) \tag{20}$$

$$\text{with} \qquad \theta s = \sum_Z \sigma_Z \cdot N_\mathrm{Z,s} \tag{21}$$

$$k_\mathrm{d,Z} = 1/\tau_\mathrm{d,Z} \tag{22}$$

$$\text{with} \qquad \tau_\mathrm{d,Z} = \nu_\mathrm{0,Z} \cdot \exp\left(-E_\mathrm{des,Z}/RT\right) \tag{23}$$

$$k_\mathrm{bss,Z} = \frac{2D_\mathrm{b,Z}}{d_\mathrm{ss} + d_\mathrm{b}} \cdot (1 - \theta_\mathrm{ss}) \tag{24}$$

$$\text{with} \qquad \theta ss = \sum_Z \sigma_Z \cdot N_\mathrm{Z,ss} \tag{25}$$

$$k_\mathrm{ssb,Z} = \frac{2D_\mathrm{b,Z}}{(\delta_\mathrm{Z} \cdot (d_\mathrm{ss} + d_\mathrm{b}))} \tag{26}$$

$$\tag{27}$$

where $\alpha_\mathrm{s,0}$ is a surface accommodation coefficient, w is the mean thermal velocity, and D_b is a bulk diffusion coefficient. θ_s and θ_ss are the surface coverages of sorption and quasi-static surface layer, respectively. Note that k_ssb was set to zero in this study. For $\nu_\mathrm{0,Z}$, we chose $1{\times}12$ s^{-1} according to Knopf et al. (2024).

S5.6 Model geometry

To account for particle growth and shrinkage, the model layer geometry of bulk and surface layers is updated during every model time step. The geometry of the gas phase in the flat bed reactor stays constant.

$$V_{\mathrm{b}} = V_{\mathrm{m,C}^e} \cdot N_{\mathrm{C}^e\mathrm{,b}} + V_{\mathrm{m,C}^b} \cdot N_{\mathrm{C}^b\mathrm{,b}} \tag{28}$$

$$d_{\mathrm{b}} = \frac{3}{4\pi N_{\mathrm{p}}} \cdot V_{\mathrm{b}}^{1/3} \tag{29}$$

$$d_{\mathrm{ss}} = \delta_{\mathrm{C}} \tag{30}$$

$$V_{\mathrm{ss}} = N_{\mathrm{p}} \cdot 4\pi/3 \cdot (d_{\mathrm{ss}} + d_{\mathrm{b}})^3 - V_{\mathrm{b}} \tag{31}$$

$$d_{\mathrm{s}} = \delta_{\mathrm{NO2}} \tag{32}$$

$$V_{\mathrm{s}} = N_{\mathrm{p}} \cdot 4\pi/3 \cdot (d_{\mathrm{s}} + d_{\mathrm{ss}} + d_{\mathrm{b}})^3 - V_{\mathrm{ss}} + V_{\mathrm{b}} \tag{33}$$

$$A_{\mathrm{b}} = 4\pi(d_{\mathrm{b}}/2)^2 \cdot N_{\mathrm{p}} \tag{34}$$

$$A_{\mathrm{ss}} = 4\pi(d_{\mathrm{ss}} + d_{\mathrm{b}}/2)^2 \cdot N_{\mathrm{p}} \tag{35}$$

$$A_{\mathrm{s}} = 4\pi(d_{\mathrm{s}} + d_{\mathrm{ss}} + d_{\mathrm{b}}/2)^2 \cdot N_{\mathrm{p}} \tag{36}$$

$$\tag{37}$$

S6 Global optimization results

The kinetic model parameters determined from repeated global optimization of KM-GAP-CARBON with the reference mechanism are tabulated in Tables S1 and S2, and the process of obtaining these parameters is described in Section S4. We obtained a fit ensemble with N=50 individual fits that achieve high correlation with the experimental data; Figs. 2-4 in the main text show the results of the best-fitting parameter set. Some parameters in the fit ensemble, such as the initial fraction of C^e atoms, f_{edge}, as well as the reaction rate coefficients of reactions R1a, R1b, and R2b, are tightly constrained by the model. Some parameters are not as tightly constrained, but occupy a limited range within the fitting parameter space, such as the activation energies of reactions R1a, R1b, R2a, and R2b. Other parameters, such as the reaction rate coefficients of reactions R4 and R5, show co-dependence. This means that their numerical value is not constrained by the global fit, but they stand in relation to each other so that if one were uniquely determined, the other were determined as well (Berkemeier et al., 2017). Other parameters are not uniquely constrained by global optimization and appear strongly scattered within their fitting boundaries (Table S5). This either means they are unimportant for the model outcome, or their effect can be compensated by another process or parameter. In general, the uniqueness of the global fit could be improved by additional experimental data or narrower *a priori* constraint of kinetic parameters.

S7 Sensitivity analysis and degree of rate control

The degree to which a reaction of rate k_i affects the global reaction rate r, $X_{\mathrm{rc},i}$, can be calculated according to Eq. (38) after Campbell (1994), which is equivalent to a normalized sensitivity coefficient (Saltelli et al., 2007; Berkemeier et al., 2013).

$$X_{\mathrm{rc},i} = \frac{\log r}{\log k_i} \tag{38}$$

S8 Dependence on O_2 and H_2O concentrations

The global reaction rate was also determined as a function of O_2 and H_2O concentration by Messerer et al. (2006), which is shown in Figs. S7 and S8. The model using reference mechanism A is able to describe the observed trend in O_2 well, with the exception of the experiments at lowered NO_2 concentrations. The effect of H_2O on soot oxidation cannot be described with our reference mechanism due to the lack of reactions with H_2O as oxidant, however, the model agrees well with the data at all H_2O concentrations except $[H_2O] = 0$.

We also fitted the data using KM-GAP-CARBON with extended mechanism B in which the chemistry that is observed for NO_2 and O_2 is expanded to water vapor, H_2O. We find that, while the model fit equality is overall improved through the addition of more, flexible model parameters (Fig. S9), the H_2O-dependence is still not fully captured (Fig. S10) and the overall contribution of H_2O as oxidant likely overestimated (Fig. S3), contradicting previous studies (Zouaoui et al., 2014). This is likely due to the lack of independent data for oxidation with H_2O in the absence of NO_2 and especially O_2.

Note, however, that while we are confident that the data provided in Messerer et al. (2006) constrains the temperature-dependent chemistry of NO_2 well, the constraint on the individual contributions of O_2 and H_2O is less well constraint. Due to the relatively free parameter space during global optimization, the measurable catalytic effect of small amounts H_2O, and the lack of data that investigates H_2O in the absence of O_2 and NO_2, the model likely overestimates the contribution of H_2O (Fig. S3b). We thus suggest and plan to perform further experiments investigating the role of H_2O in the oxidation of soot nanoparticles in this temperature range.

We suggest and plan to perform further experiments investigating the role of H_2O in the oxidation of soot nanoparticles in this temperature range. In these experiments, the determination of individual contributions of NO_2, O_2 and H_2O could be facilitated by probing each oxidant individually. A markedly different reaction rate in the absence of water is known and may be due to a catalytic effect on the NO_2 + soot reaction (Jacquot et al., 2002; Zouaoui et al., 2014), possibly involving the formation of HONO (Kleffmann et al., 1999; Arens et al., 2001) or HNO_3 (Jeguirim et al., 2005) that is not represented in the simplified reaction mechanism presented in this study and will be subject of follow-up studies.

S9 Supplementary Figures

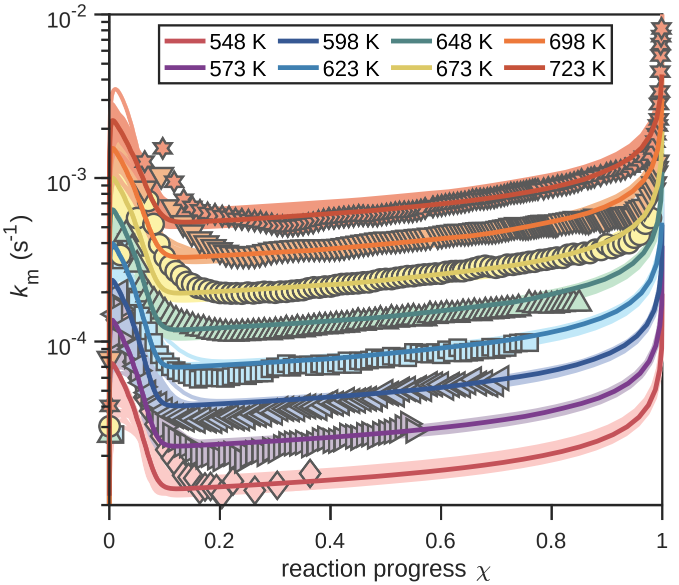

Figure S1. Mass-based, first-order reaction rate coefficient k_m as a function of reaction progress ξ. Model outputs (lines) are compared with experimental data (markers; Messerer et al., 2006) for a range of temperatures T = 548-723 K. Darker colored lines represent the globally best-fitting kinetic parameter set and lighter colored lines indicate all other fits within the fit ensemble.

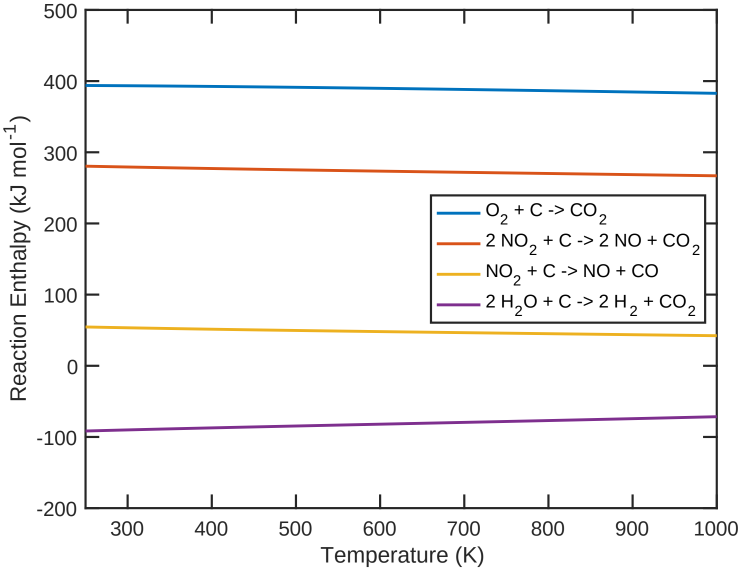

Figure S2. Temperature dependence of the reaction enthalpies (ΔH_r) of the sum reactions denoted in the figure legend and used for Fig. 6 in the main text. ΔH_r are derived from standard formation enthalpies, calculated using polynomials tabulated in the NASA Glenn thermodynamic database (McBride et al., 2002)

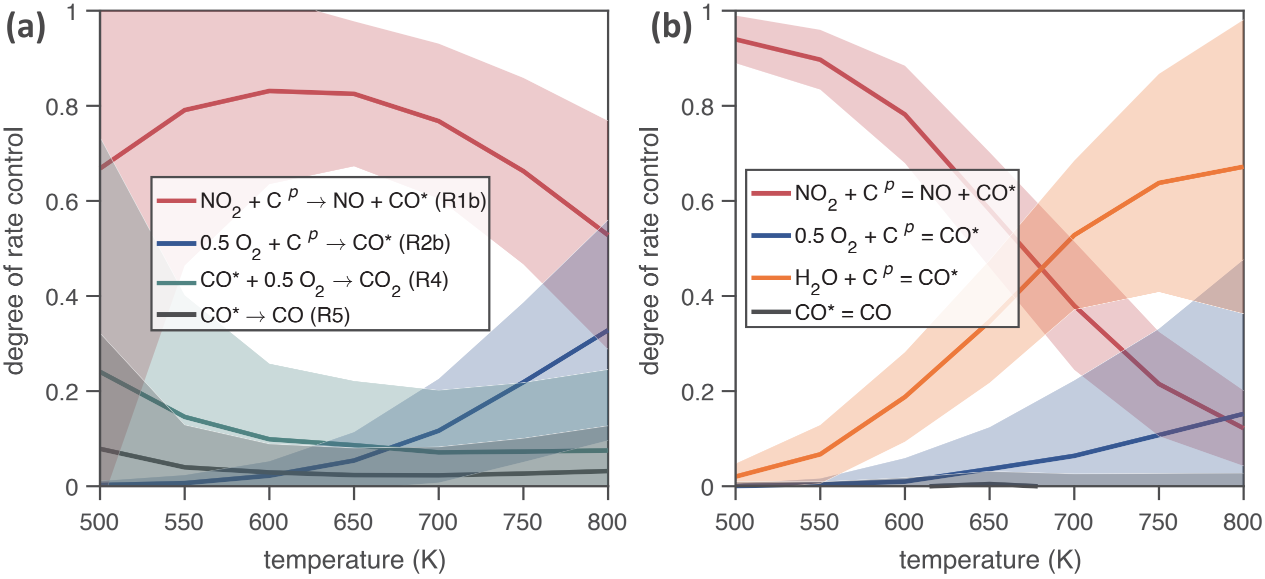

Figure S3. Analysis of the degree of rate control (DRC) in KM-GAP-CARBON for the global rate of soot loss as a function of temperature (experiments 1-8 in Table S3), (a) using the reference mechanism A with 10-fold increased NO_2 mixing ratios ($N = 50$ fit ensemble), and (b) using the extended reference mechanism B ($N = 20$ fit ensemble). Solid lines indicate the mean of the fit ensemble, while shadings indicate two standard deviations around the mean. Displayed are the four reactions with the highest DRC.

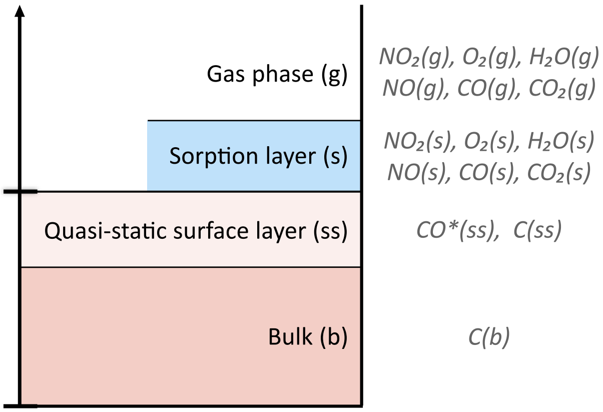

Figure S4. Model compartments and structure of the kinetic multi-layer model of gas-particle interactions for high-temperature multiphase chemistry of carbon nanoparticles (KM-GAP-CARBON) and chemical species from the reference mechanism A residing in the four model compartments.

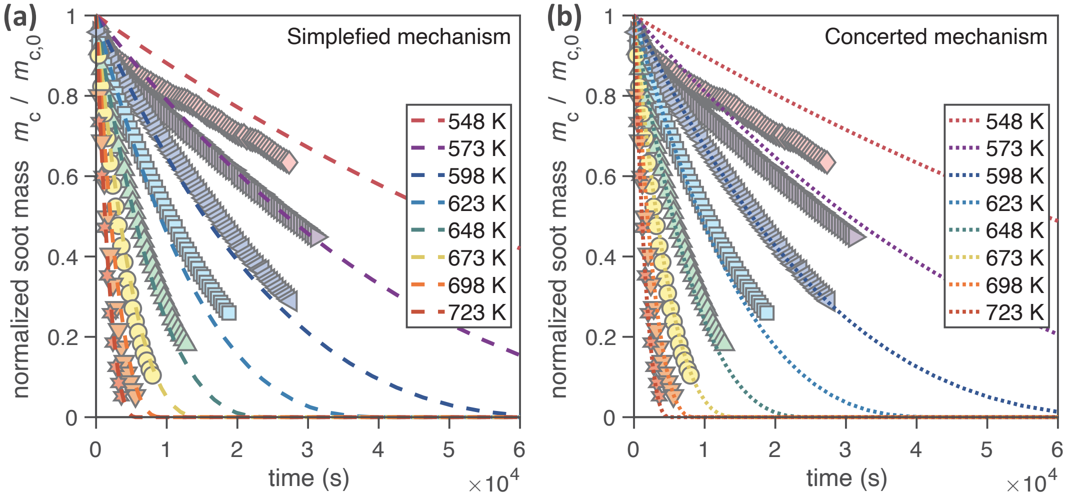

Figure S5. Comparison of the performance of simplified model schemes in the high-temperature soot gasification by NO_2 in the presence of O_2 and water vapor: (a) single ROI mechanism, (b) concerted mechanism. After global optimization, both model schemes yield a similar correlation with experimental data. Note that neither of the mechanisms resolves soot nanostructure, which leads to inferior model-experiment correlation compared to the single ROI + nanostructure mechanism discussed in the main text.

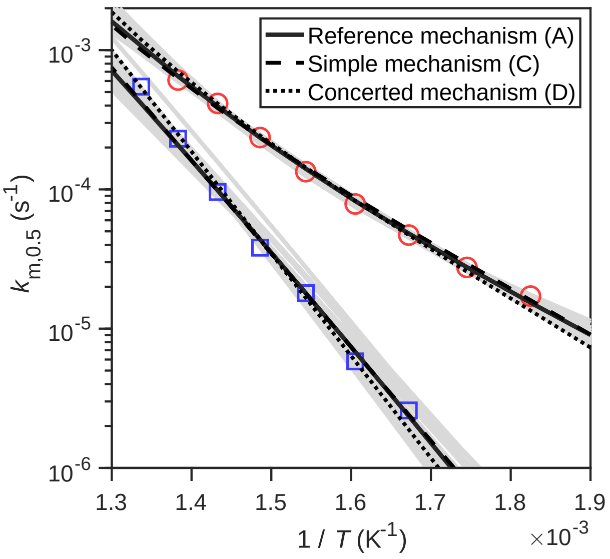

Figure S6. Comparison of different model schemes in the Arrhenius plot of $k_{\mathrm{m},0.5}$, the global, mass-based, pseudo first-order rate coefficient of soot gasification at 50 % reaction progress (ξ). All three model schemes, reference mechanism A (solid black line), simplified mechanism C (dashed black line), and concerted mechanism D (dotted black line) describe the experimental well and within the ranges of the fit ensemble obtained with multiple global optimizations using the reference mechanism (light grey lines). The non-linearity of the dependence of logarithmic $k_{\mathrm{m},0.5}$ against inverse temperature is less pronounced in the concerted model scheme, yielding a slightly worse model-experiment correlation.

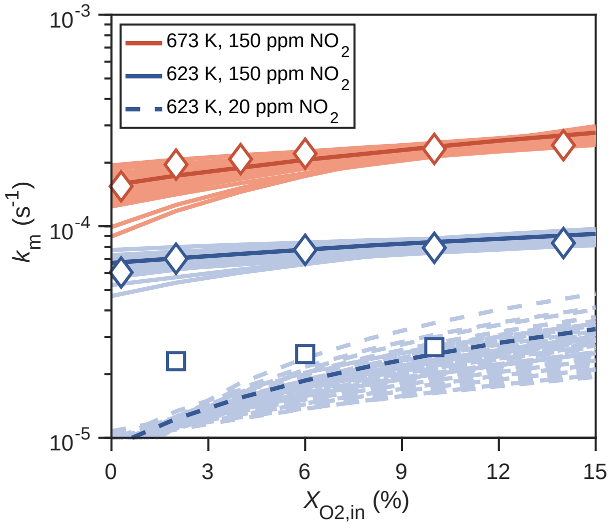

Figure S7. Influence of oxygen mole fraction (X_{O2}) on mass-based first-order rate coefficient at 50 % reaction progress, $k_{m,0.5}$. Markers represent experimental data from Messerer et al. (2006); lines represent model results (KM-GAP-CARBON with reference mechanism), where darker colors represents the globally best-fitting kinetic parameter set and lighter colors indicate all other fits within the fit ensemble. All experiments were performed with 3 % H_2O and NO_2 as indicated in the figure legend (Tab. S3).

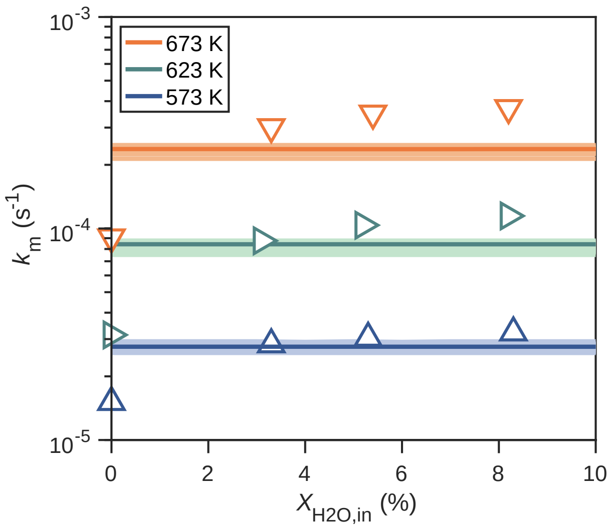

Figure S8. Influence of water mole fraction (X_{H2O}) on mass-based first-order rate coefficient at 50 % reaction progress, $k_{m,0.5}$. Markers represent experimental data from Messerer et al. (2006); lines represent model results (KM-GAP-CARBON with reference mechanism), where darker colors represents the globally best-fitting kinetic parameter set and lighter colors indicate all other fits within the fit ensemble. All experiments were performed with 150 ppb of NO_2 and 10 % O_2 (Tab. S3).

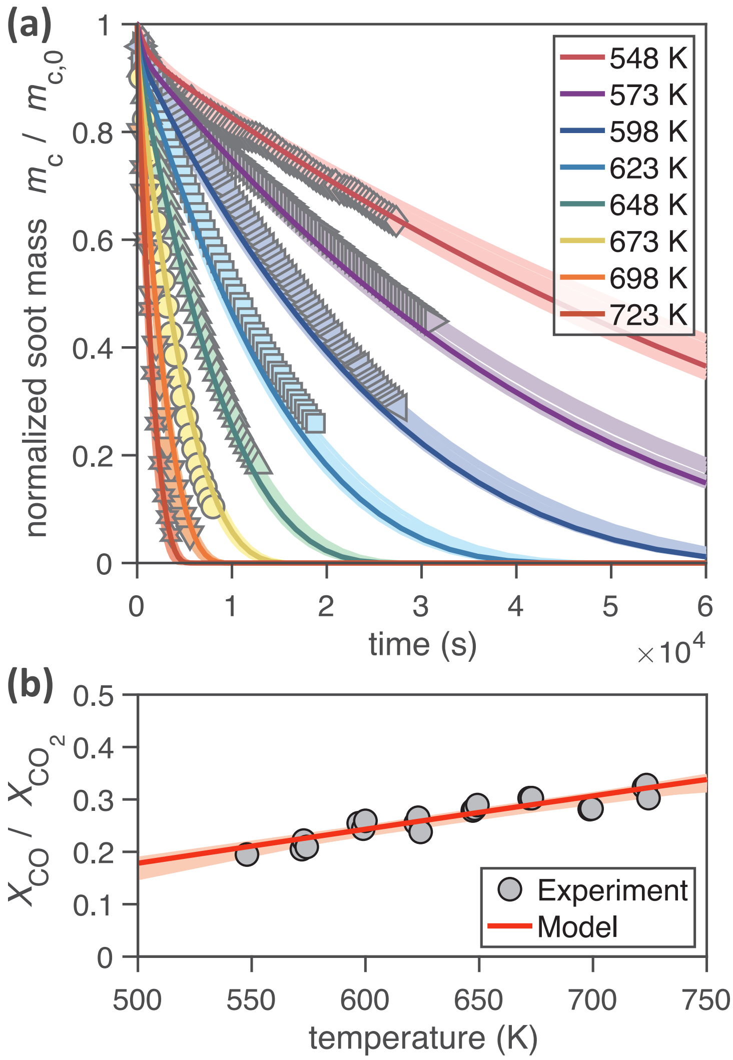

Figure S9. Carbon nanoparticle oxidation and gasification by NO_2 (150 ppm), O_2 (10 %), and H_2O (3 %). (a) Decay of normalized soot mass over time for eight experiments at T = 548-723 K and (b) the ratio of the mole fractions (X) of CO and CO_2 in the reactor outflow as a function of temperature. Experimental data (markers; Messerer et al., 2006) is compared with KM-GAP-CARBON model output using the extended reference mechanism B (lines). Thick solid lines represent the globally best-fitting kinetic parameter set, while shadings indicate the variability within the fit ensemble.

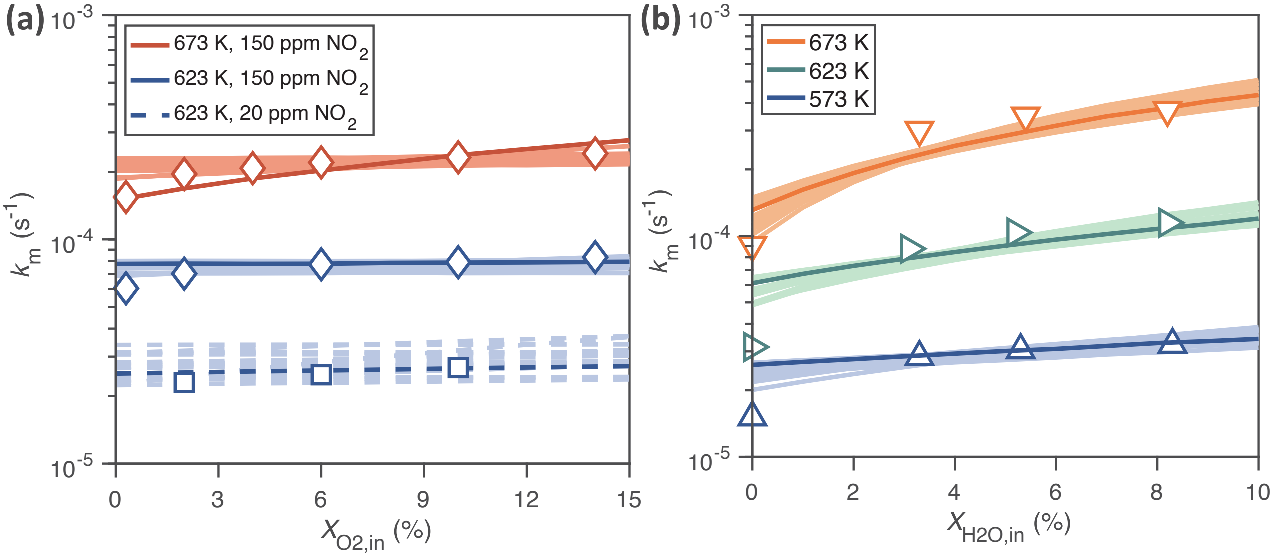

Figure S10. Influence of (a) oxygen mole fraction (X_{O2}) and (b) water mole fraction (X_{H2O}) on mass-based first-order rate coefficient at 50 % reaction progress, $k_{m,0.5}$ using KM-GAP-CARBON with the extended reference mechanism B. Markers represent experimental data from Messerer et al. (2006); lines represent model results, where darker colors represents the globally best-fitting kinetic parameter set and lighter colors indicate all other fits within the fit ensemble. For experimental conditions, refer to experiments 20-37 in Tab. S3 and the figure legend.

S10 Supplementary Tables

Table S1. Ensemble of 50 kinetic parameter sets of the KM-GAP-CARBON model using the reference mechanism A and obtained during global optimization to experimental data in Messerer et al. (2006) - Part 1: Fraction of edge-like carbons and reaction rate coefficients at 623 K.

Fit # / Unit	f_{edge} $cm^2\ s^{-1}$	$k_{1a,\ 623K}$ $cm^2\ s^{-1}$	$k_{1b,\ 623K}$ $cm^2\ s^{-1}$	$k_{2a,\ 623K}$ $cm^2\ s^{-1}$	$k_{2b,\ 623K}$ $cm^2\ s^{-1}$	$k_{3,\ 623K}$ $cm^2\ s^{-1}$	$k_{4,\ 623K}$ s^{-1}	$k_{5,\ 623K}$
1	0.329	4.01E-11	1.21E-12	1.21E-16	3.62E-16	8.33E-17	9.00E-13	4.52E-02
2	0.359	2.96E-11	1.08E-12	1.18E-16	4.41E-16	2.20E-18	9.38E-13	4.72E-02
3	0.359	2.96E-11	1.08E-12	1.18E-16	4.41E-16	2.20E-18	9.38E-13	4.72E-02
4	0.319	5.25E-11	1.27E-12	1.22E-16	3.36E-16	7.58E-15	1.55E-13	7.59E-03
5	0.336	4.65E-11	1.22E-12	1.26E-16	3.49E-16	1.00E-18	1.54E-13	7.51E-03
6	0.314	4.94E-11	1.24E-12	1.18E-16	3.99E-16	4.93E-17	2.07E-13	1.03E-02
7	0.327	5.26E-11	1.33E-12	1.34E-16	3.00E-16	8.45E-18	1.34E-13	6.55E-03
8	0.388	2.73E-11	1.11E-12	1.04E-16	4.00E-16	1.02E-15	3.68E-13	1.83E-02
9	0.341	6.73E-11	1.32E-12	1.34E-16	3.01E-16	1.64E-14	7.79E-14	3.69E-03
10	0.324	5.09E-11	1.33E-12	1.62E-16	2.37E-16	2.52E-16	1.60E-13	7.88E-03
11	0.293	8.68E-11	1.27E-12	8.74E-17	3.85E-16	4.73E-14	1.19E-13	5.77E-03
12	0.321	6.32E-11	1.36E-12	2.07E-16	2.18E-16	1.21E-14	1.11E-13	5.37E-03
13	0.335	3.92E-11	1.10E-12	9.70E-17	3.93E-16	3.07E-17	5.23E-13	2.59E-02
14	0.373	3.47E-11	1.25E-12	1.16E-16	2.88E-16	1.52E-18	1.20E-13	5.80E-03
15	0.355	2.98E-11	1.01E-12	1.05E-16	5.19E-16	6.77E-14	5.65E-13	2.81E-02
16	0.330	4.25E-11	1.31E-12	1.52E-16	2.03E-16	5.36E-18	3.35E-13	1.67E-02
17	0.332	3.72E-11	1.21E-12	1.25E-16	3.15E-16	1.09E-18	3.66E-13	1.85E-02
18	0.318	7.81E-11	1.39E-12	2.16E-16	2.08E-16	1.00E-14	9.27E-14	4.44E-03
19	0.367	3.17E-11	1.27E-12	4.09E-16	1.63E-16	4.43E-15	2.19E-13	1.08E-02
20	0.384	2.97E-11	1.27E-12	1.84E-16	2.12E-16	1.57E-14	1.14E-13	5.51E-03
21	0.369	4.70E-11	1.30E-12	1.38E-16	2.65E-16	1.27E-18	7.32E-14	3.44E-03
22	0.342	1.40E-10	1.47E-12	5.52E-16	1.45E-16	1.05E-16	6.10E-14	2.82E-03
23	0.354	3.04E-11	1.21E-12	1.25E-16	2.61E-16	4.06E-14	1.67E-13	8.18E-03
24	0.326	3.68E-11	1.26E-12	2.26E-16	1.95E-16	2.72E-14	4.31E-13	2.15E-02
25	0.327	3.58E-11	1.32E-12	1.22E-16	2.81E-16	3.60E-16	1.76E-13	8.55E-03
26	0.352	2.56E-11	1.31E-12	6.02E-15	1.35E-16	4.76E-15	4.16E-13	2.04E-02
27	0.336	3.99E-11	1.43E-12	2.19E-16	1.40E-16	4.66E-18	1.42E-13	6.87E-03
28	0.429	1.81E-11	1.20E-12	1.13E-16	3.78E-16	2.25E-17	7.54E-14	3.55E-03
29	0.363	3.81E-11	1.13E-12	1.22E-16	3.19E-16	8.79E-15	1.22E-13	5.84E-03
30	0.382	2.81E-11	1.10E-12	1.83E-16	3.68E-16	2.60E-16	1.64E-13	8.17E-03
31	0.347	3.62E-11	1.24E-12	1.75E-16	2.36E-16	4.23E-18	4.16E-13	2.20E-02
32	0.308	6.45E-11	1.39E-12	9.47E-17	2.73E-16	1.22E-15	1.94E-13	9.82E-03
33	0.358	2.85E-11	1.17E-12	2.18E-16	2.68E-16	3.37E-17	3.90E-13	2.02E-02
34	0.353	3.38E-11	1.22E-12	1.95E-16	3.00E-16	1.43E-14	2.68E-13	1.28E-02
35	0.328	3.33E-11	1.09E-12	1.16E-16	3.22E-16	5.27E-18	5.73E-13	2.95E-02

Table S1. Continued.

Fit # / Unit	f_{edge} $cm^2\ s^{-1}$	$k_{1a,\ 623K}$ $cm^2\ s^{-1}$	$k_{1b,\ 623K}$ $cm^2\ s^{-1}$	$k_{2a,\ 623K}$ $cm^2\ s^{-1}$	$k_{2b,\ 623K}$ $cm^2\ s^{-1}$	$k_{3,\ 623K}$ $cm^2\ s^{-1}$	$k_{4,\ 623K}$ s^{-1}	$k_{5,\ 623K}$
36	0.367	2.81E-11	1.23E-12	1.60E-16	2.89E-16	8.87E-14	1.85E-13	8.88E-03
37	0.352	3.79E-11	1.21E-12	1.99E-16	3.08E-16	3.08E-14	1.63E-13	8.04E-03
38	0.318	5.92E-11	1.17E-12	1.12E-16	3.24E-16	9.75E-18	1.52E-13	7.15E-03
39	0.352	3.20E-11	1.26E-12	3.05E-16	1.52E-16	2.58E-16	1.95E-13	9.33E-03
40	0.331	4.54E-11	1.29E-12	2.18E-16	3.42E-16	1.73E-15	1.73E-13	8.15E-03
41	0.312	2.59E-11	1.46E-12	5.45E-15	1.83E-16	5.14E-14	5.22E-13	2.58E-02
42	0.330	3.43E-11	1.27E-12	4.00E-16	2.72E-16	7.77E-15	3.61E-13	1.77E-02
43	0.355	3.26E-11	1.18E-12	1.59E-16	3.32E-16	5.08E-18	2.31E-13	1.05E-02
44	0.346	2.76E-11	9.33E-13	7.85E-17	5.24E-16	2.00E-15	7.14E-13	3.45E-02
45	0.320	5.19E-11	1.36E-12	2.41E-16	2.01E-16	5.78E-18	1.81E-13	9.82E-03
46	0.325	4.14E-11	1.17E-12	7.44E-17	3.73E-16	8.42E-17	3.70E-13	2.03E-02
47	0.343	3.96E-11	1.41E-12	3.37E-15	1.42E-16	4.65E-16	1.25E-13	5.65E-03
48	0.340	2.90E-11	1.33E-12	5.62E-15	1.77E-16	3.48E-17	6.29E-13	2.88E-02
49	0.308	3.94E-11	1.29E-12	1.25E-15	2.48E-16	8.81E-18	2.73E-13	1.36E-02
50	0.334	3.75E-11	8.85E-13	8.50E-17	6.57E-16	1.08E-15	1.96E-13	8.80E-03

Table S2. Ensemble of 50 kinetic parameter sets of the KM-GAP-CARBON model using the reference mechanism A and obtained during global optimization to experimental data in Messerer et al. (2006) - Part 2: Arrhenius activation energies and fitness value R of the model fit in comparison to experimental data.

Fit # / Unit	$\mathbf{E_{A,1a}}$ kJ mol^{-1}	$\mathbf{E_{A,1b}}$ kJ mol^{-1}	$\mathbf{E_{A,2a}}$ kJ mol^{-1}	$\mathbf{E_{A,2b}}$ kJ mol^{-1}	$\mathbf{E_{A,3}}$ kJ mol^{-1}	$\mathbf{E_{A,4}}$ kJ mol^{-1}	$\mathbf{E_{A,5}}$ kJ mol^{-1}	$\boldsymbol{R}$
1	81.9	76.3	160.2	130.3	130.5	74.1	64.6	3.952
2	78.1	73.2	155.6	128.2	62.5	84.8	75.3	3.988
3	78.1	73.2	155.6	128.2	62.5	84.8	75.3	3.988
4	79.1	76.3	160.2	135.5	124.5	81.9	72.3	3.991
5	92.6	73.6	154.0	138.1	120.8	73.2	63.3	4.001
6	86.6	75.7	150.6	130.5	116.6	74.5	64.8	4.045
7	94.5	77.9	162.2	135.4	73.7	82.4	72.9	4.078
8	91.8	76.8	187.7	120.8	83.7	73.4	63.8	4.082
9	80.2	76.0	160.0	140.1	60.4	95.6	86.8	4.098
10	98.7	77.7	161.8	145.6	76.8	71.6	61.7	4.104
11	83.1	71.2	163.4	142.3	87.1	93.8	84.7	4.110
12	89.7	76.9	145.1	151.6	72.8	86.7	77.4	4.132
13	85.1	69.2	160.5	136.8	128.6	83.1	74.0	4.142
14	86.6	75.3	182.5	136.7	104.9	89.0	79.8	4.161
15	82.3	71.7	164.1	123.5	81.3	82.4	72.9	4.176
16	79.7	76.4	162.6	153.6	60.4	109.7	100.5	4.189
17	84.5	76.3	161.4	139.3	104.4	97.0	86.3	4.196
18	90.2	76.8	137.5	155.5	67.0	71.6	61.4	4.212
19	84.6	77.1	137.0	154.9	93.1	76.3	66.7	4.268
20	88.4	76.4	164.7	145.3	127.8	109.1	100.3	4.290
21	67.6	74.0	170.9	143.3	84.6	97.5	89.0	4.293
22	80.0	79.0	142.2	156.7	92.5	90.1	81.2	4.316
23	78.1	76.2	157.5	154.3	71.4	75.6	65.4	4.395
24	83.5	76.5	136.5	158.4	62.5	87.5	78.0	4.399
25	78.7	75.4	192.1	134.2	145.8	98.3	90.6	4.471
26	74.2	79.8	148.4	154.1	138.3	92.6	83.6	4.528
27	91.3	80.1	182.0	156.0	92.0	72.7	63.0	4.569
28	72.5	80.3	180.6	124.3	141.8	66.0	55.0	4.582
29	93.4	73.4	155.0	149.4	139.1	81.7	73.8	4.603
30	85.4	71.8	127.6	135.5	65.2	92.8	83.8	4.651
31	89.6	77.1	141.2	149.8	87.3	109.1	99.9	4.679
32	89.6	81.1	189.0	133.4	146.6	81.6	70.0	4.707
33	93.0	75.2	137.5	142.9	93.5	116.7	108.1	4.707
34	79.2	79.7	135.7	135.6	70.2	95.4	87.5	4.776
35	79.5	67.7	159.9	145.6	81.3	85.3	76.7	4.783

Table S2. Continued.

Fit # / Unit	$\mathbf{E_{A,1a}}$ kJ mol^{-1}	$\mathbf{E_{A,1b}}$ kJ mol^{-1}	$\mathbf{E_{A,2a}}$ kJ mol^{-1}	$\mathbf{E_{A,2b}}$ kJ mol^{-1}	$\mathbf{E_{A,3}}$ kJ mol^{-1}	$\mathbf{E_{A,4}}$ kJ mol^{-1}	$\mathbf{E_{A,5}}$ kJ mol^{-1}	**RMSE**
36	75.8	78.5	156.3	137.6	63.4	91.2	86.8	4.783
37	90.9	77.8	147.2	127.8	104.2	115.9	105.2	4.801
38	75.0	73.3	158.8	150.8	101.1	111.1	102.1	4.816
39	75.0	75.3	126.9	160.9	65.1	80.8	72.7	4.837
40	98.1	78.1	167.0	124.3	149.4	78.1	71.1	4.842
41	69.0	82.4	186.4	139.2	127.3	76.9	67.4	4.862
42	70.6	74.9	129.9	142.9	134.7	103.3	95.6	4.873
43	91.1	76.4	145.4	127.6	106.3	72.3	66.6	4.994
44	76.6	61.5	163.3	136.6	135.0	73.0	62.6	4.995
45	81.4	73.9	132.3	157.6	114.0	100.2	87.6	5.069
46	88.2	71.6	169.7	139.9	134.8	79.0	63.5	5.078
47	60.6	79.6	173.1	148.2	125.3	114.7	102.0	5.089
48	71.7	82.2	149.2	140.1	139.5	112.8	105.1	5.222
49	83.4	74.9	123.0	152.3	95.7	79.0	69.9	5.255
50	75.8	60.3	165.8	126.6	103.0	93.2	85.5	5.256

Table S3. Experimental conditions for 37 experiments conducted in Messerer et al. (2006) and used for global optimization of kinetic model parameters in this study.

Exp. #	$[NO_2]_0$ ppm	$[O_2]_0$ %	$[H_2O]_0$ %	$[NO]_0$ ppm	Temperature K	Exp. #	$[NO_2]_0$ ppm	$[O_2]_0$ %	$[H_2O]_0$ %	$[NO]_0$ ppm	Temperature K
1	150	10	3	45	548	20	150	2	3	0	623
2	150	10	3	45	573	21	150	6	3	0	623
3	150	10	3	45	598	22	150	0.3	3	0	673
4	150	10	3	45	623	23	150	2	3	0	673
5	150	10	3	45	648	24	150	6	3	0	673
6	150	10	3	45	673	25	150	14	3	0	673
7	150	10	3	45	698	26	150	10	0	0	573
8	150	10	3	45	723	27	150	10	3.3	0	573
9	0	10	3	0	598	28	150	10	5.4	0	573
10	0	10	3	0	623	29	150	10	8.2	0	573
11	0	10	3	0	648	30	150	10	0	0	623
12	0	10	3	0	673	31	150	10	3.1	0	623
13	0	10	3	0	698	32	150	10	5.1	0	623
14	0	10	3	0	723	33	150	10	8.2	0	623
15	0	10	3	0	748	34	150	10	0	0	673
16	20	2	3	0	623	35	150	10	3.3	0	673
17	20	6	3	0	623	36	150	10	5.4	0	673
18	20	10	3	0	623	37	150	10	8.2	0	673
19	150	0.3	3	0	623						

Table S4. Kinetic parameters of the KM-GAP-CARBON model that remained fixed during global optimization.

Parameter	Value	Unit	Description
$m_{C,0}$	7.5×10^{-3}	g	Initial soot mass loading
V_g	21	cm^3	Volume of flat bed reactor
$\dot{V}_{flow}$	5	NL min^{-1}	Reactor feed gas flow
$[NO_2]_{g,0}$	0–150	ppm	Reactor feed gas concentration of NO_2
$[NO]_{g,0}$	45	ppm	Reactor feed gas concentration of NO
$[O_2]_{g,0}$	0–15	%	Reactor feed gas concentration of O_2
$[H_2O]_{g,0}$	0–10	%	Reactor feed gas concentration of H_2O
d_p	1×10^{-6}	cm	Diameter of soot primary particle
ρ_p	1.5	g cm^{-3}	Density of soot primary particle
σ_{site}	1.3×10^{-15}	cm^2	Effective surface area of sorption site
$\nu_{0,Z}$	1×10^{12}	s^{-1}	Attempt frequency of desorption of Z
$H_{ads,Z}$	15	kJ mol^{-1}	Enthalpy of physisorption of Z
$\alpha_{s,0,Z}$	1		Surface accommodation coefficient of Z on adsorbate free surface

Table S5. Kinetic parameters of the KM-GAP-CARBON model varied within boundaries during global optimization.

Parameter	Lower Boundary	Upper Boundary	Unit	Description
f_{edge}	0.05	0.95	–	Initial fraction of edge-like carbon atoms
$k_{1a, 623K}$	1×10^{-13}	1×10^{-9}	$cm^2\ s^{-1}$	Reaction rate coefficient of reaction R1a at 623 K
$k_{1b, 623K}$	1×10^{-14}	1×10^{-10}	$cm^2\ s^{-1}$	Reaction rate coefficient of reaction R1b at 623 K
$k_{2a, 623K}$	1×10^{-19}	1×10^{-14}	$cm^2\ s^{-1}$	Reaction rate coefficient of reaction R2a at 623 K
$k_{2b, 623K}$	1×10^{-19}	1×10^{-14}	$cm^2\ s^{-1}$	Reaction rate coefficient of reaction R2b at 623 K
$k_{3, 623K}$	1×10^{-18}	1×10^{-13}	$cm^2\ s^{-1}$	Reaction rate coefficient of reaction R3a at 623 K
$k_{4, 623K}$	1×10^{-17}	1×10^{-12}	$cm^2\ s^{-1}$	Reaction rate coefficient of reaction R4 at 623 K
$k_{5, 623K}$	1×10^{-4}	1	s^{-1}	Reaction rate coefficient of reaction R5 at 623 K
$E_{A,1a}$	60	120	kJ mol^{-1}	Arrhenius activation energy of reaction R1a
$E_{A,1b}$	60	120	kJ mol^{-1}	Arrhenius activation energy of reaction R1b
$E_{A,2a}$	120	210	kJ mol^{-1}	Arrhenius activation energy of reaction R2a
$E_{A,2b}$	120	210	kJ mol^{-1}	Arrhenius activation energy of reaction R2b
$E_{A,3}$	60	150	kJ mol^{-1}	Arrhenius activation energy of reaction R3a
$E_{A,4}$	60	120	kJ mol^{-1}	Arrhenius activation energy of reaction R4
$E_{A,5}$	60	120	kJ mol^{-1}	Arrhenius activation energy of reaction R5

Table S6. Ensemble of 20 kinetic parameter sets of the KM-GAP-CARBON model using the extended reference mechanism B and obtained during global optimization to experimental data in Messerer et al. (2006) - Part 1: Fraction of edge-like carbons and reaction rate coefficients at 623 K.

Fit # / Unit	f_{edge}	$k_{1a, 623K}$ $cm^2\ s^{-1}$	$k_{1b, 623K}$ $cm^2\ s^{-1}$	$k_{2a, 623K}$ $cm^2\ s^{-1}$	$k_{2b, 623K}$ $cm^2\ s^{-1}$	$k_{3a, 623K}$ $cm^2\ s^{-1}$	$k_{3b, 623K}$ $cm^2\ s^{-1}$	$k_{4, 623K}$ $cm^2\ s^{-1}$	$k_{5, 623K}$ $cm^2\ s^{-1}$	$k_{6, 623K}$ $cm^2\ s^{-1}$	$k_{7, 623K}$ s^{-1}
1	0.317	3.65E-11	1.08E-12	8.04E-17	3.21E-17	1.66E-12	1.89E-10	9.64E+00	4.35E-17	1.01E-15	1.18E-13
2	0.363	3.11E-11	9.17E-13	9.68E-17	1.37E-19	4.34E-10	5.69E-10	2.88E+01	1.50E-17	1.32E-15	6.73E-13
3	0.335	3.08E-11	9.99E-13	6.90E-17	3.10E-19	3.37E-12	1.02E-10	2.56E+01	7.83E-17	1.15E-15	9.84E-10
4	0.388	2.31E-11	8.72E-13	1.10E-16	2.22E-16	1.13E-13	2.27E-10	1.18E+01	3.10E-17	8.45E-16	1.60E-11
5	0.338	3.38E-11	9.52E-13	8.36E-17	4.96E-18	5.12E-13	1.35E-10	6.80E+00	3.24E-17	1.37E-15	2.75E-13
6	0.325	3.58E-11	8.83E-13	3.14E-17	2.86E-18	5.13E-11	2.79E-13	9.02E+00	1.24E-16	1.59E-15	4.52E-10
7	0.388	2.54E-11	1.03E-12	5.17E-17	5.97E-17	9.22E-12	7.86E-11	4.64E+00	1.08E-16	8.31E-16	3.40E-11
8	0.307	3.69E-11	1.02E-12	6.33E-17	3.31E-17	2.46E-13	9.02E-10	4.64E+01	5.78E-17	1.23E-15	3.91E-11
9	0.347	2.56E-11	7.66E-13	4.79E-17	9.77E-17	2.01E-12	3.87E-11	4.79E+01	1.14E-16	1.46E-15	2.27E-09
10	0.350	2.80E-11	9.66E-13	6.23E-17	8.38E-18	8.22E-11	3.51E-10	1.75E+01	1.40E-16	1.18E-15	5.14E-12
11	0.390	2.30E-11	1.06E-12	6.08E-17	1.06E-18	1.42E-12	5.63E-11	3.41E+00	1.23E-16	8.64E-16	2.70E-11
12	0.350	2.59E-11	8.61E-13	3.43E-17	5.20E-18	8.01E-12	1.39E-10	6.95E+00	1.39E-16	1.16E-15	5.08E-13
13	0.370	2.38E-11	9.12E-13	8.56E-17	8.13E-17	1.50E-11	6.29E-10	3.21E+01	3.48E-17	1.12E-15	2.33E-13
14	0.385	2.02E-11	8.97E-13	4.37E-19	2.37E-17	4.41E-11	5.67E-11	3.05E+00	2.99E-16	1.09E-15	7.58E-12
15	0.348	3.14E-11	9.27E-13	4.68E-17	5.87E-17	8.43E-13	3.06E-12	8.66E+00	1.10E-16	9.36E-16	4.18E-10
16	0.341	3.24E-11	9.35E-13	1.12E-16	9.11E-19	9.46E-11	2.90E-10	1.48E+01	3.74E-18	1.39E-15	1.14E-11
17	0.324	3.65E-11	1.15E-12	5.97E-17	2.15E-17	1.95E-11	2.26E-10	1.13E+01	8.87E-17	7.97E-16	1.43E-13
18	0.395	2.24E-11	9.75E-13	7.67E-17	6.31E-17	1.17E-11	3.56E-11	2.75E+00	1.02E-16	8.42E-16	5.08E-11
19	0.303	4.46E-11	1.03E-12	6.06E-17	2.04E-19	1.10E-11	1.77E-12	2.81E+00	1.04E-16	1.20E-15	1.35E-10
20	0.329	4.07E-11	1.09E-12	5.27E-17	1.12E-17	7.33E-11	8.11E-10	4.17E+01	1.66E-16	8.81E-16	4.24E-13

Table S7. Ensemble of 20 kinetic parameter sets of the KM-GAP-CARBON model using the extended reference mechanism B and obtained during global optimization to experimental data in Messerer et al. (2006) - Part 2: Arrhenius activation energies and fitness value R of the model fit in comparison to experimental data.

Fit # / Unit	$\mathbf{E_{A,1a}}$ kJ mol^{-1}	$\mathbf{E_{A,1b}}$ kJ mol^{-1}	$\mathbf{E_{A,2a}}$ kJ mol^{-1}	$\mathbf{E_{A,2b}}$ kJ mol^{-1}	$\mathbf{E_{A,3a}}$ kJ mol^{-1}	$\mathbf{E_{A,3b}}$ kJ mol^{-1}	$\mathbf{E_{A,4}}$ kJ mol^{-1}	$\mathbf{E_{A,5}}$ kJ mol^{-1}	$\mathbf{E_{A,6}}$ kJ mol^{-1}	$\mathbf{E_{A,7}}$ kJ mol^{-1}	$\boldsymbol{R}$
1	87.5	66.8	161.5	148.4	67.7	111.3	101.7	73.4	134.0	141.6	2.742
2	86.6	65.7	164.7	166.6	83.7	84.0	74.0	109.2	128.0	80.9	2.819
3	89.2	65.4	166.9	175.3	138.8	79.9	81.2	129.2	133.7	93.5	2.821
4	88.0	61.1	155.5	126.3	108.6	98.0	89.3	115.1	136.8	127.4	2.824
5	93.8	66.7	174.2	193.3	84.7	81.7	73.0	71.5	121.9	78.5	2.883
6	84.3	65.9	203.1	204.0	104.1	104.7	100.5	81.0	118.8	110.8	2.892
7	95.5	70.1	195.1	139.9	103.0	79.5	75.8	61.4	133.2	111.3	2.896
8	85.9	70.8	186.4	177.9	60.8	100.0	91.6	83.2	117.4	133.2	2.910
9	100.1	64.7	186.0	162.1	109.3	101.6	83.8	90.8	110.6	92.9	2.911
10	101.2	62.8	176.2	147.0	130.3	129.0	120.3	85.9	139.0	98.8	2.914
11	81.8	72.9	192.7	180.4	74.6	144.1	135.1	73.1	130.2	144.7	2.918
12	94.7	60.2	199.7	155.7	138.7	102.3	92.9	76.3	139.7	69.3	2.939
13	77.1	64.8	170.2	160.5	139.6	114.0	104.8	71.4	114.3	104.2	2.945
14	83.2	63.9	125.5	160.6	77.4	111.2	100.1	146.7	132.8	75.9	2.975
15	90.0	66.8	198.6	123.9	103.7	91.3	115.5	73.1	135.9	125.9	2.977
16	76.9	65.4	149.4	206.1	86.6	134.7	124.5	142.0	125.9	141.0	2.988
17	79.6	69.4	181.9	178.4	69.2	139.9	131.6	81.3	135.7	123.3	2.990
18	81.6	65.5	166.9	123.1	71.3	127.1	118.2	86.2	149.0	128.3	3.010
19	72.3	70.2	186.8	194.3	110.0	93.1	90.7	84.3	131.8	100.5	3.049
20	88.5	67.8	180.8	200.0	112.0	138.9	128.6	67.9	131.8	143.6	3.095

Table S8. List of symbols.

Symbol	Description	Unit
$\alpha_{\mathrm{s,0,Z}}$	surface accommodation coefficient of Z on adsorbate free surface	
A_{s}	Surface area of sorption layer compartment	cm^2
A_{ss}	Surface area of quasi-static surface layer compartment	cm^2
A_{b}	Surface area of bulk compartment	cm^2
d_{s}	Thickness / diameter of sorption layer compartment	cm
d_{ss}	Thickness / diameter of quasi-static surface layer compartment	cm
d_{b}	Thickness / diameter of bulk layer compartment	cm
δ_{Z}	Effective molecular diameter of Z	cm
$D_{\mathrm{b,Z}}$	Bulk diffusion coefficient of Z	$\mathrm{cm}^2\ \mathrm{s}^{-1}$
$\mathrm{H}_{\mathrm{ads,Z}}$	Enthalpy of physisorption of Z	$\mathrm{kJ\ mol}^{-1}$
$k_{\mathrm{a,Z}}$	First-order adsorption rate coefficient of Z	$\mathrm{cm\ s}^{-1}$
$k_{\mathrm{d,Z}}$	First-order desorption rate coefficient of Z	s^{-1}
$k_{\mathrm{ssb,Z}}$	First-order rate coefficients for surface-bulk transport of Z	s^{-1}
$k_{\mathrm{bss,Z}}$	First-order rate coefficients for bulk-surface transport of Z	$\mathrm{cm\ s}^{-1}$
N_{p}	Number concentration of particles	cm^{-3}
$N_{\mathrm{Z,g}}$	Number of molecules of species Z in gas phase compartment	
$N_{\mathrm{Z,s}}$	Number of molecules of species Z in sorption layer compartment	
$N_{\mathrm{Z,ss}}$	Number of molecules of species Z in quasi-static surface layer compartment	
$N_{\mathrm{Z,b}}$	Number of molecules of species Z in bulk compartment	
R	Fitness value of model fit in comparison with experimental data	
σ_{Z}	Effective molecular surface cross section of Z	cm^2
θ_{ss}	Surface coverage of sorption layer compartment	
$\nu_{\mathrm{0,Z}}$	Attempt frequency of desorption of Z	s^{-1}
V_{g}	Volume of gas phase compartment	cm^3
V_{s}	Volume of sorption layer compartment	cm^3
V_{ss}	Volume of quasi-static surface layer compartment	cm^3
V_{b}	Volume of bulk compartment	cm^3
$\dot{V}_{\mathrm{flow}}$	Volumetric flow rate	$\mathrm{cm}^3\ \mathrm{s}^{-1}$
w_{Z}	Mean thermal velocity of Z	$\mathrm{cm\ s}^{-1}$
$[\mathrm{Z}]_{\mathrm{g}}$	Gas-phase concentration of species Z	cm^{-3}
$[\mathrm{Z}]_{\mathrm{s}}$	Sorption layer concentration of species Z	cm^{-3}
$[\mathrm{Z}]_{\mathrm{ss}}$	Quasi-static surface layer concentration of species Z	cm^{-3}
$[\mathrm{Z}]_{\mathrm{b}}$	Bulk concentration of species Z	cm^{-3}

References

Alfè, M., Apicella, B., Barbella, R., Rouzaud, J.-N., Tregrossi, A., and Ciajolo, A.: Structure–property relationship in nanostructures of young and mature soot in premixed flames, Proc. Combust., 32, 697–704, https://doi.org/10.1016/j.proci.2008.06.193, 2009.

Arens, F., Gutzwiller, L., Baltensperger, U., Gäggeler, H. W., and Ammann, M.: Heterogeneous Reaction of NO2 on Diesel Soot Particles, Environ. Sci. Technol., 35, 2191–2199, https://doi.org/10.1021/es000207s, 2001.

Berkemeier, T., Huisman, A. J., Ammann, M., Shiraiwa, M., Koop, T., and Pöschl, U.: Kinetic regimes and limiting cases of gas uptake and heterogeneous reactions in atmospheric aerosols and clouds: a general classification scheme, Atmos. Chem. Phys., 13, 6663–6686, https://doi.org/10.5194/acp-13-6663-2013, 2013.

Berkemeier, T., Ammann, M., Krieger, U. K., Peter, T., Spichtinger, P., Pöschl, U., Shiraiwa, M., and Huisman, A. J.: Technical note: Monte Carlo genetic algorithm (MCGA) for model analysis of multiphase chemical kinetics to determine transport and reaction rate coefficients using multiple experimental data sets, Atmos. Chem. Phys., 17, 8021–8029, https://doi.org/10.5194/acp-17-8021-2017, 2017.

Berkemeier, T., Takeuchi, M., Eris, G., and Ng, N. L.: Kinetic modeling of formation and evaporation of secondary organic aerosol from NO3 oxidation of pure and mixed monoterpenes, Atmos. Chem. Phys., 20, 15 513–15 535, https://doi.org/10.5194/acp-20-15513-2020, 2020.

Berkemeier, T., Mishra, A., Mattei, C., Huisman, A. J., Krieger, U. K., and Pöschl, U.: Ozonolysis of Oleic Acid Aerosol Revisited: Multiphase Chemical Kinetics and Reaction Mechanisms, ACS Earth Space Chem., 5, 3313–3323, https://doi.org/10.1021/acsearthspacechem.1c00232, 2021.

Campbell, C. T.: Future Directions and Industrial Perspectives Micro- and macro-kinetics: Their relationship in heterogeneous catalysis, Top. Catal., 1, 353–366, https://doi.org/10.1007/BF01492288, 1994.

Chen, S. G., Yang, R. T., Kapteijn, F., and Moulijn, J. A.: A new surface oxygen complex on carbon: toward a unified mechanism for carbon gasification reactions, Ind. Eng. Chem. Res., 32, 2835–2840, https://doi.org/10.1021/ie00023a054, 1993.

Dobbins, R., Fletcher, R., and Chang, H.-C.: The evolution of soot precursor particles in a diffusion flame, Combust. Flame, 115, 285–298, https://doi.org/10.1016/S0010-2180(98)00010-8, 1998.

Ergun, S.: Kinetics of the Reaction of Carbon with Carbon Dioxide, J. Phys. Chem., 60, 480–485, https://doi.org/10.1021/j150538a022, 1956.

Jacquot, F., Logie, V., J.-F. Brilhac, Brilhac, J.-F., P. Gilot, and Gilot, P.: Kinetics of the oxidation of carbon black by NO2: Influence of the presence of water and oxygen, Carbon, 40, 335–343, https://doi.org/10.1016/s0008-6223(01)00103-8, 2002.

Jeguirim, M., Tschamber, V., Brilhac, J.-F., Brilhac, J., and Ehrburger, P.: Oxidation mechanism of carbon black by NO2: Effect of water vapour, Fuel, 84, 1949–1956, https://doi.org/10.1016/j.fuel.2005.03.026, 2005.

Kleffmann, J., H. Becker, K., Lackhoff, M., and Wiesen, P.: Heterogeneous conversion of NO2 on carbonaceous surfaces, Phys. Chem. Chem. Phys., 1, 5443–5450, https://doi.org/10.1039/A905545B, 1999.

Knopf, D. A., Ammann, M., Berkemeier, T., Pöschl, U., and Shiraiwa, M.: Desorption lifetimes and activation energies influencing gas–surface interactions and multiphase chemical kinetics, Atmos. Chem. Phys., 24, 3445–3528, https://doi.org/10.5194/acp-24-3445-2024, 2024.

Krüger, M., Mishra, A., Spichtinger, P., Pöschl, U., and Berkemeier, T.: A numerical compass for experiment design in chemical kinetics and molecular property estimation, J. Cheminf., 16, 34, https://doi.org/10.1186/s13321-024-00825-0, 2024.

Li, C. and Brown, T. C.: Carbon oxidation kinetics from evolved carbon oxide analysis during temperature-programmed oxidation, Carbon, 39, 725–732, https://doi.org/10.1016/S0008-6223(00)00189-5, 2001.

Mansurov, Z. A.: Soot Formation in Combustion Processes (Review), Combust. Explos. Shock Waves, 41, 727–744, https://doi.org/10.1007/s10573-005-0083-2, 2005.

McBride, B. J., Zehe, M. J., and Gordon, S.: NASA Glenn coefficients for calculating thermodynamic properties of individual species, National Aeronautics and Space Administration, John H. Glenn Research Center, https://ntrs.nasa.gov/citations/20020085330, 2002.

Messerer, A., Schmid, H.-J., Knab, C., Pöschl, U., and Nießner, R.: Erhöhung der Abscheidung ultrafeiner Dieselruß-partikeln durch Mikrokugelbeschichtung auf metallträgerbasierten Katalysatorstrukturen, Chem. Ing. Tech., 76, 1092–1096, https://doi.org/10.1002/cite.200403418, 2004.

Messerer, A., Niessner, R., and Pöschl, U.: Comprehensive kinetic characterization of the oxidation and gasification of model and real diesel soot by nitrogen oxides and oxygen under engine exhaust conditions: Measurement, Langmuir–Hinshelwood, and Arrhenius parameters, Carbon, 44, 307–324, https://doi.org/10.1016/j.carbon.2005.07.017, 2006.

Michelsen, H.: Probing soot formation, chemical and physical evolution, and oxidation: A review of in situ diagnostic techniques and needs, Proc. Combust., 36, 717–735, https://doi.org/10.1016/j.proci.2016.08.027, 2017.

Muckenhuber, H. and Grothe, H.: The heterogeneous reaction between soot and NO2 at elevated temperature, Carbon, 44, 546–559, https://doi.org/10.1016/j.carbon.2005.08.003, 2006.

Ouf, F.-X., Bourrous, S., Fauvel, S., Kort, A., Lintis, L., Nuvoli, J., and Yon, J.: True density of combustion emitted particles: A comparison of results highlighting the influence of the organic contents, J. Aero. Sci., 134, 1–13, https://doi.org/10.1016/j.jaerosci.2019.04.007, 2019.

Reilly, P. T. A., Gieray, R. A., Whitten, W. B., and Ramsey, J. M.: Fullerene Evolution in Flame-Generated Soot, J. Am. Chem. Soc., 122, 11 596–11 601, https://doi.org/10.1021/ja003521v, 2000.

Sabbah, H., Commodo, M., Picca, F., De Falco, G., Minutolo, P., D'Anna, A., and Joblin, C.: Molecular content of nascent soot: Family characterization using two-step laser desorption laser ionization mass spectrometry, Proc. Combust., 38, 1241–1248, https://doi.org/10.1016/j.proci.2020.09.022, 2021.

Saltelli, A., Ratto, M., Andres, T., Campolongo, F., Cariboni, J., Gatelli, D., Saisana, M., and Tarantola, S.: Introduction to Sensitivity Analysis, John Wiley & Sons, Ltd, https://doi.org/10.1002/9780470725184, 2007.

Shiraiwa, M., Pfrang, C., Koop, T., and Pöschl, U.: Kinetic multi-layer model of gas-particle interactions in aerosols and clouds (KM-GAP): linking condensation, evaporation and chemical reactions of organics, oxidants and water, Atmos. Chem. Phys., 12, 2777–2794, https://doi.org/10.5194/acp-12-2777-2012, 2012.

Smith, G. P., Golden, D. M., and et al.: GRI-Mech 3.0, http://www.me.berkeley.edu/gri_mech/, retrieved 2024.

Stanmore, B., Brilhac, J., and Gilot, P.: The oxidation of soot: a review of experiments, mechanisms and models, Carbon, 39, 2247–2268, https://doi.org/10.1016/S0008-6223(01)00109-9, 2001.

Zouaoui, N., Labaki, M., and Jeguirim, M.: Diesel soot oxidation by nitrogen dioxide, oxygen and water under engine exhaust conditions: Kinetics data related to the reaction mechanism, C. R. Chim., 17, 672–680, https://doi.org/10.1016/j.crci.2013.09.004, 2014.
